# Supplementary material for: Ancient Maltese genomes and the genetic geography of Neolithic Europe
Source: Curr Biol. 2022 Jun 20;32(12):2668–2680.e6. doi: 10.1016/j.cub.2022.04.069 (PMC9245899; doi:10.1016/j.cub.2022.04.069)
Supplement: Document S1. Figures S1–S7 [file mmc1.pdf]

Current Biology, Volume *32*

Supplemental Information

Ancient Maltese genomes and the genetic geography of Neolithic Europe

Bruno Ariano, Valeria Mattiangeli, Emily M. Breslin, Eóin W. Parkinson, T. Rowan McLaughlin, Jess E. Thompson, Ronika K. Power, Jay T. Stock, Bernardette Mercieca-Spiteri, Simon Stoddart, Caroline Malone, Shyam Gopalakrishnan, Lara M. Cassidy, and Daniel G. Bradley

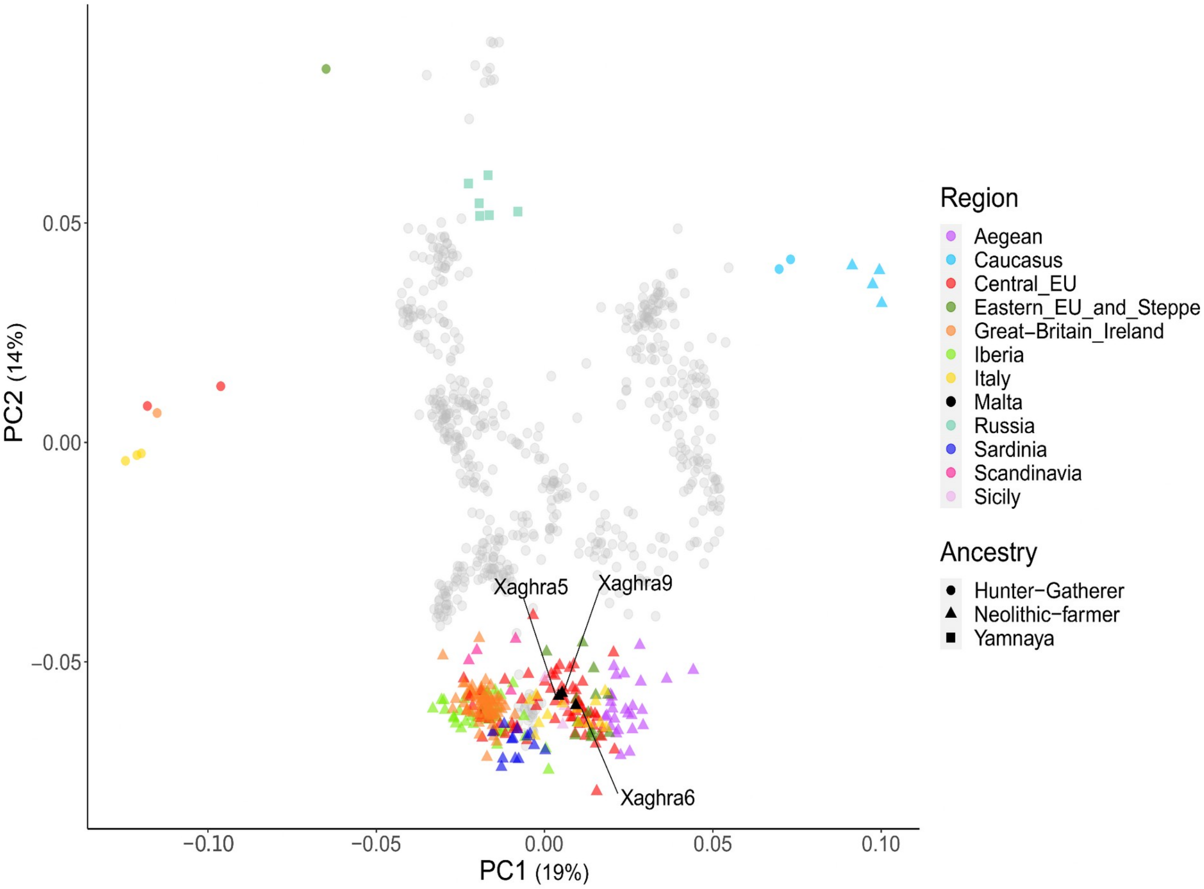

Figure S1: PCA for ancient Maltese and other ancient European populations. **Related to Star Methods and Data S2.** Maltese genomes as well as other published Neolithic and Mesolithic genomes were projected onto a principal component analysis plot using the Human Origins dataset as a modern reference. For each principal component the percentage of variance explained is indicated in parentheses. The ancient Maltese samples group together with Central and Southern European Neolithic genomes.

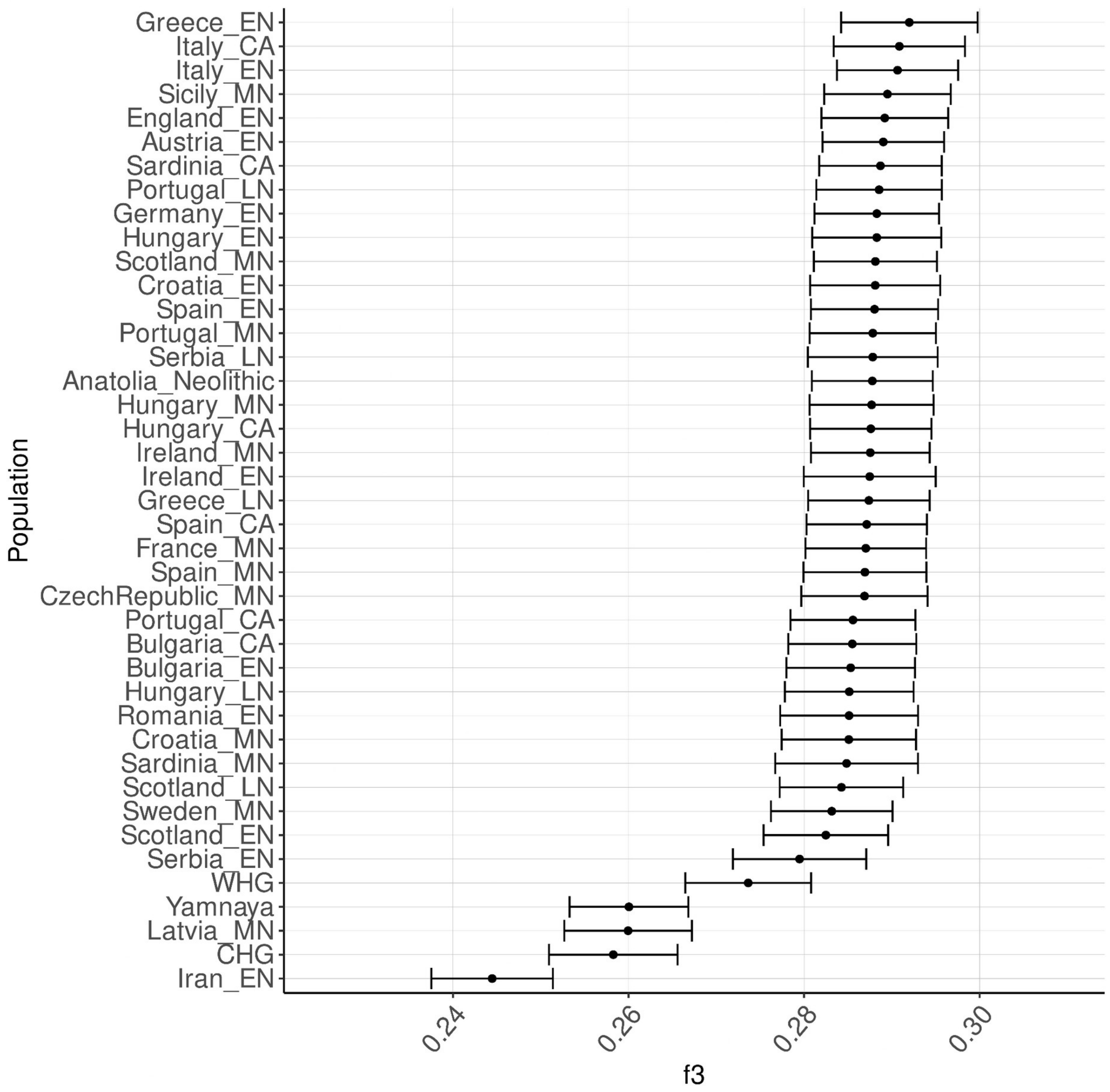

Figure S2: Shared drift measured using the outgroup-f3 statistics. **Related to Star Methods and Data S2F.** The results using outgroup-f3 statistics represented by circles with error bars in the form (Mbuti; X, Malta Neolithic) show the Maltese being closer to early Neolithic individuals from Greece and the Italian peninsula. (EN=Early Neolithic, MN=Middle Neolithic, LN=Late Neolithic, CA=Copper Age, WHG=Western hunter-gatherer, CHG=Caucasus hunter-gatherer, EHG=Eastern hunter-gatherer). The Anatolian Neolithic population is represented by individuals from Marmara, Barcın.

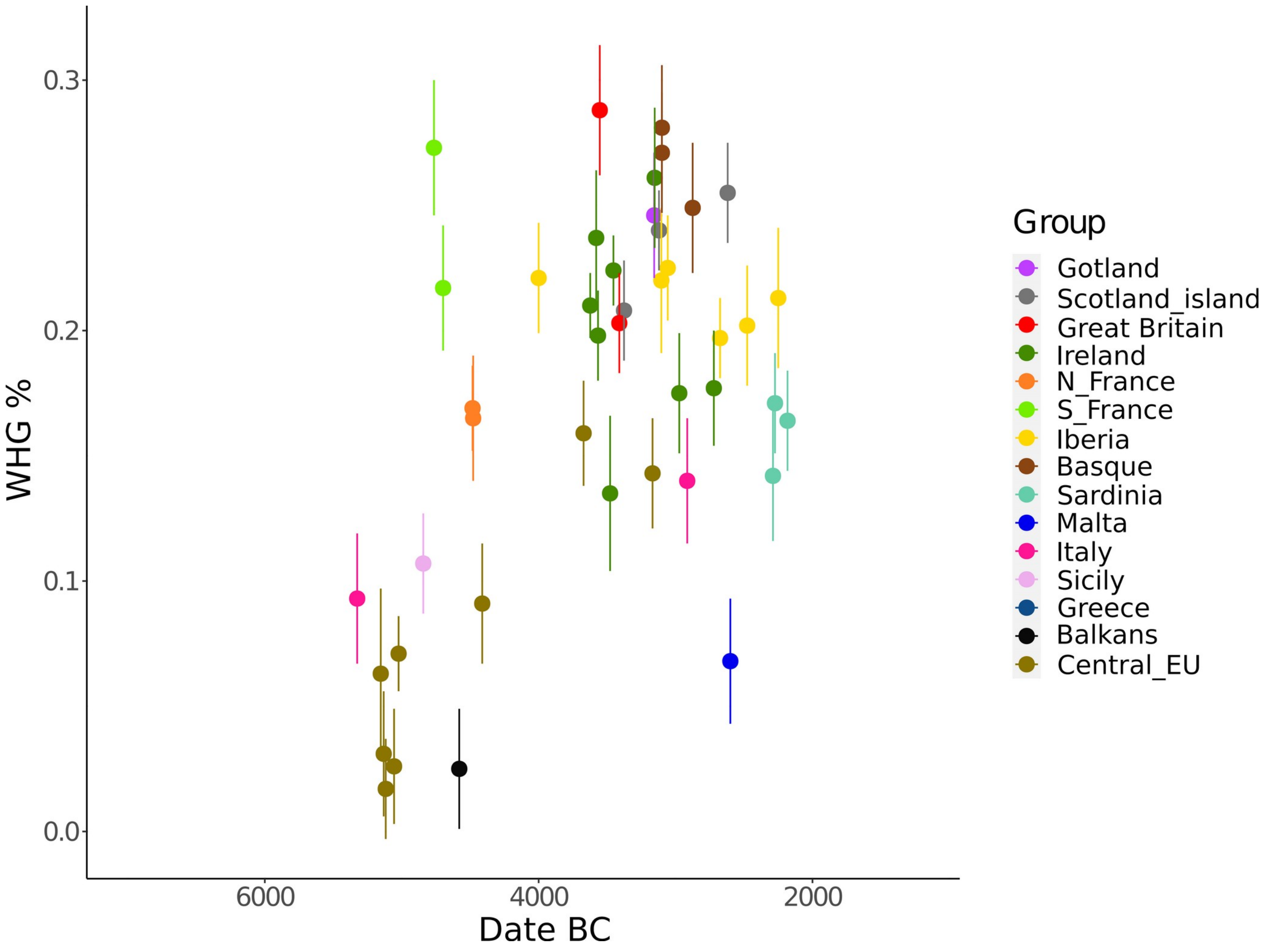

Figure S3: Temporal distribution of hunter-gatherer ancestry in Neolithic Europeans. Related to Star Methods and Data S2E. qpAdm was used to measure the quantity of Western Hunter-gatherer (WHG) populations, represented by the individuals KO1 and Loschbour, present in European Neolithic populations. Each point represents with its error bar a group with at least 2 individuals from an archaeological site and time period. The WHG percentages are reported with 95% error bars.

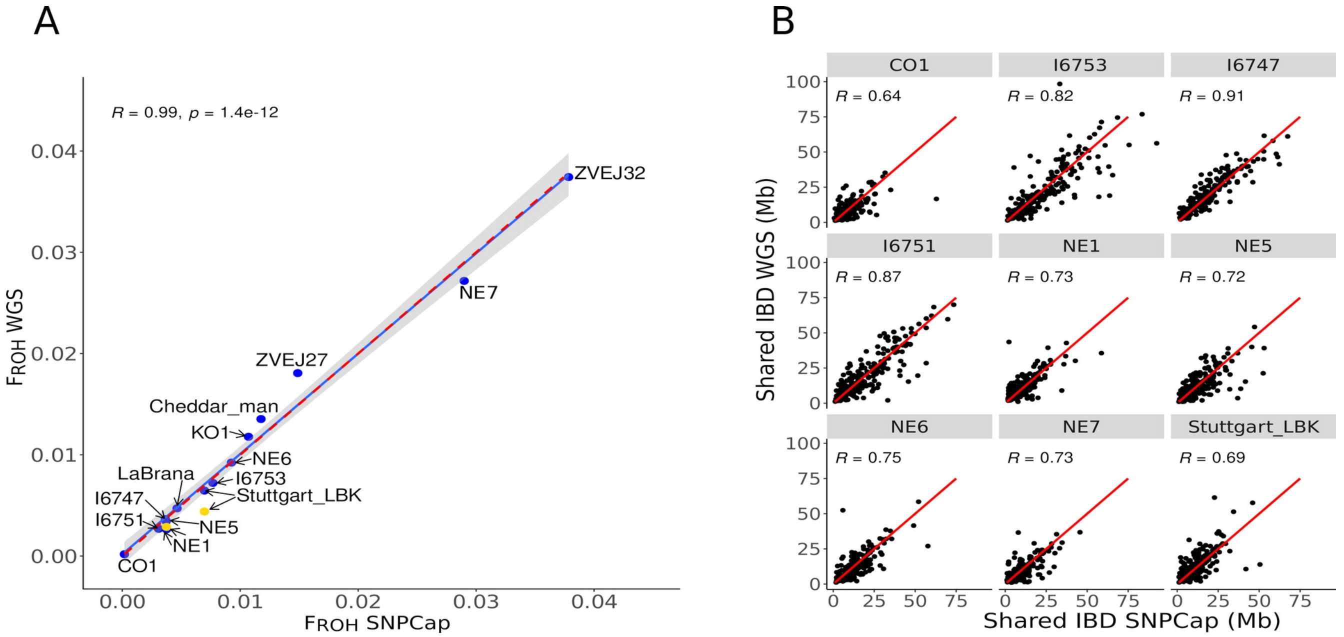

Figure S4: Comparisons of IBD and F_{ROH} estimates from imputed WGS and SNP captured data. Related to Star Methods and Data S1B, S1D. (A) F_{ROH} were compared between imputations of WGS and SNP captured data (respectively "F_{ROH} WGS" and "F_{ROH} SNPCap") where these are available from the same samples. These correlate with a P-value lower than and the regression line (coloured blue) error margins overlap with the 1:1 plot (red line). Also two yellow coloured points denote where three genomes (NE1, Stuttgart_LBK) also had WGS F_{ROH} estimates available from high coverage SNP calls - these are plotted Vs F_{ROH} SNPCap. (B) Plots of IBD sharing values involving each of nine Neolithic samples for which WGS and SNP capture - based estimates are available. For each, total estimates of the genome shared with all other Neolithic samples is plotted alternately using the two different data sets. The WGS and SNP-derived values correlate significantly (each at $p < 10^{-15}$) and vary around the 1:1 plotline, drawn in red.

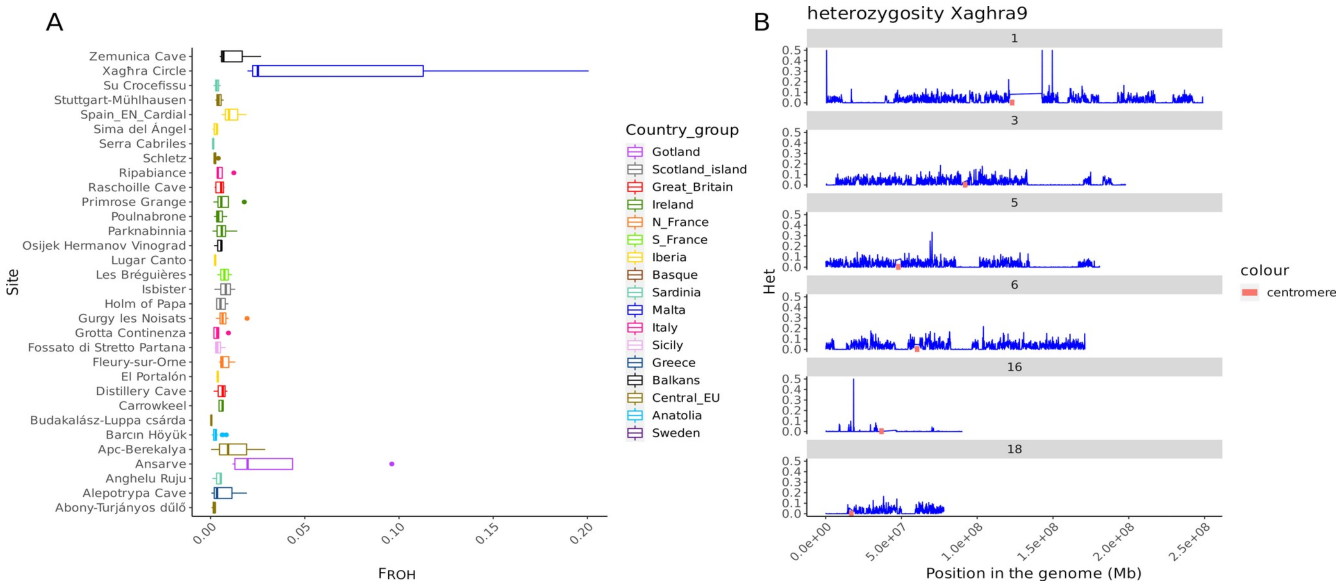

Figure S5: Inbreeding F_{ROH} coefficient estimates and chromosome heterozygosity plots for Xaghra9. Related to Figure 2A and Data S1D. (A) F_{ROH} coefficients within sites with a minimum of 3 individuals. The Maltese possess the highest median inbreeding coefficient followed by values from Gotland. (B) The average heterozygosity is plotted for Xaghra9 using a window size of 100 kb and illustrates examples of long genome tracts of homozygosity in selected chromosomes.

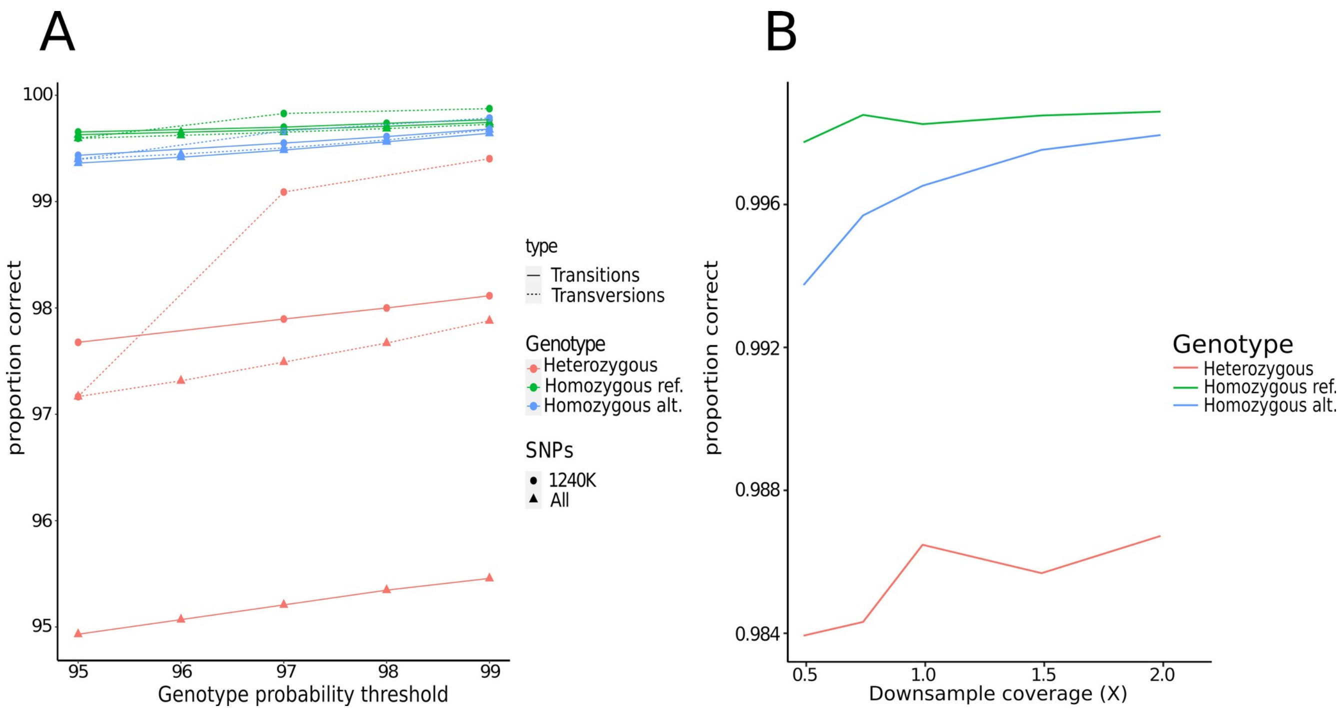

Figure S6: Accuracy of imputed genotypes. **Related to Star Methods.** (A) Genotypes imputed from a SNP captured genome (LBK) compared against those called from its high coverage genome sequence using the software SNPSift[S43]. Heterozygous and transversion SNPs show overall lower accuracy compared to homozygous and transition imputed SNPs. The SNPs corresponding to the 1240K positions were predicted overall with higher accuracy compared to other variants. (B) Genotypes imputed from a downsampled WGS genome (LBK) also here compared against its high coverage genome sequence. Only transversions are considered in this analysis and heterozygous calls show overall less accuracy than homozygous ones. Overall the percentage of safely imputed genotypes remains high for each genotype class across different probability thresholds.

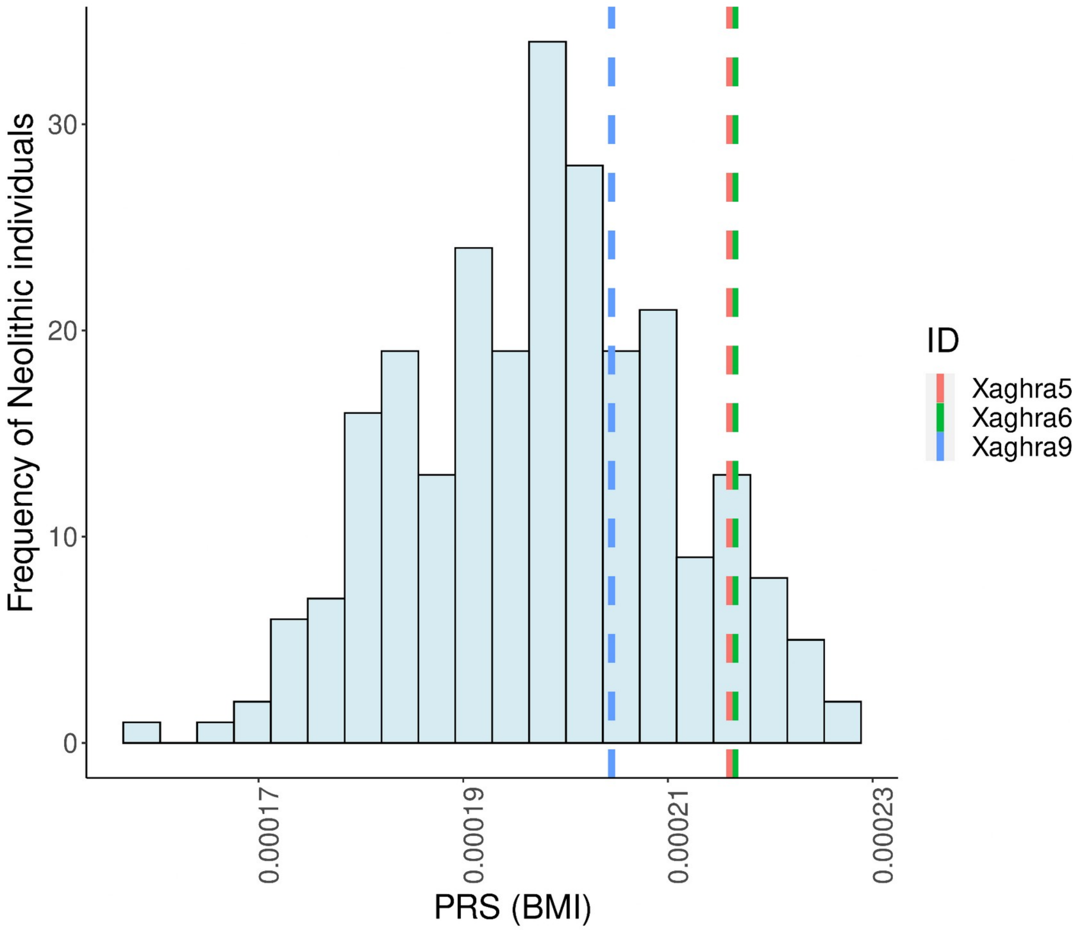

Figure S7: Body mass index in ancient Maltese. **Related to Star Methods.** Polygenic risk score (PRS) information calculated from the UK Biobank dataset was used to estimate body mass index (BMI) of the ancient Maltese together with 247 European Neolithic samples. None of the former showed extreme values.

Supplemental References

S1. Keller, A., Graefen, A., Ball, M., Matzas, M., Boisguerin, V., Maixner, F., Leidinger, P., Backes, C., Khairat, R., Forster, M., et al. (2012). New insights into the Tyrolean Iceman's origin and phenotype as inferred by whole-genome sequencing. Nat. Commun. *3*, 698.

S2. Lazaridis, I., Patterson, N., Mittnik, A., Renaud, G., Mallick, S., Kirsanow, K., Sudmant, P.H., Schraiber, J.G., Castellano, S., Lipson, M., et al. (2014). Ancient human genomes suggest three ancestral populations for present-day Europeans. Nature *513*, 409–413.

S3. Skoglund, P., Malmström, H., Omrak, A., Raghavan, M., Valdiosera, C., Günther, T., Hall, P., Tambets, K., Parik, J., Sjögren, K.-G., et al. (2014). Genomic diversity and admixture differs for Stone-Age Scandinavian foragers and farmers. Science *344*, 747–750.

S4. Gamba, C., Jones, E.R., Teasdale, M.D., McLaughlin, R.L., Gonzalez-Fortes, G., Mattiangeli, V., Domboróczki, L., Kővári, I., Pap, I., Anders, A., et al. (2014). Genome flux and stasis in a five millennium transect of European prehistory. Nat. Commun. *5*, 5257.

S5. Seguin-Orlando, A., Korneliussen, T.S., Sikora, M., Malaspinas, A.-S., Manica, A., Moltke, I., Albrechtsen, A., Ko, A., Margaryan, A., Moiseyev, V., et al. (2014). Paleogenomics. Genomic structure in Europeans dating back at least 36,200 years. Science *346*, 1113–1118.

S6. Raghavan, M., Skoglund, P., Graf, K.E., Metspalu, M., Albrechtsen, A., Moltke, I., Rasmussen, S., Stafford, T.W., Jr, Orlando, L., Metspalu, E., et al. (2014). Upper Palaeolithic Siberian genome reveals dual ancestry of Native Americans. Nature *505*, 87–91.

S7. Fu, Q., Li, H., Moorjani, P., Jay, F., Slepchenko, S.M., Bondarev, A.A., Johnson, P.L.F., Aximu-Petri, A., Prüfer, K., de Filippo, C., et al. (2014). Genome sequence of a 45,000-year-old modern human from western Siberia. Nature *514*, 445–449.

S8. Olalde, I., Allentoft, M.E., Sánchez-Quinto, F., Santpere, G., Chiang, C.W.K., DeGiorgio, M., Prado-Martinez, J., Rodríguez, J.A., Rasmussen, S., Quilez, J., et al. (2014). Derived immune and ancestral pigmentation alleles in a 7,000-year-old Mesolithic European. Nature *507*, 225–228.

S9. Mathieson, I., Lazaridis, I., Rohland, N., Mallick, S., Patterson, N., Roodenberg, S.A., Harney, E., Stewardson, K., Fernandes, D., Novak, M., et al. (2015). Genome-wide patterns of selection in 230 ancient Eurasians.

Nature *528*, 499–503.

S10. Jones, E.R., Gonzalez-Fortes, G., Connell, S., Siska, V., Eriksson, A., Martiniano, R., McLaughlin, R.L., Gallego Llorente, M., Cassidy, L.M., Gamba, C., et al. (2015). Upper Palaeolithic genomes reveal deep roots of modern Eurasians. Nat. Commun. *6*, 8912.

S11. Olalde, I., Schroeder, H., Sandoval-Velasco, M., Vinner, L., Lobón, I., Ramirez, O., Civit, S., García Borja, P., Salazar-García, D.C., Talamo, S., et al. (2015). A Common Genetic Origin for Early Farmers from Mediterranean Cardial and Central European LBK Cultures. Mol. Biol. Evol. *32*, 3132–3142.

S12. Günther, T., Valdiosera, C., Malmström, H., Ureña, I., Rodriguez-Varela, R., Sverrisdóttir, Ó.O., Daskalaki, E.A., Skoglund, P., Naidoo, T., Svensson, E.M., et al. (2015). Ancient genomes link early farmers from Atapuerca in Spain to modern-day Basques. Proc. Natl. Acad. Sci. U. S. A. *112*, 11917–11922.

S13. Allentoft, M.E., Sikora, M., Sjögren, K.-G., Rasmussen, S., Rasmussen, M., Stenderup, J., Damgaard, P.B., Schroeder, H., Ahlström, T., Vinner, L., et al. (2015). Population genomics of Bronze Age Eurasia. Nature *522*, 167–172.

S14. Hofmanová, Z., Kreutzer, S., Hellenthal, G., Sell, C., Diekmann, Y., Díez-Del-Molino, D., van Dorp, L., López, S., Kousathanas, A., Link, V., et al. (2016). Early farmers from across Europe directly descended from Neolithic Aegeans. Proc. Natl. Acad. Sci. U. S. A. *113*, 6886–6891.

S15. Mallick, S., Li, H., Lipson, M., Mathieson, I., Gymrek, M., Racimo, F., Zhao, M., Chennagiri, N., Nordenfelt, S., Tandon, A., et al. (2016). The Simons Genome Diversity Project: 300 genomes from 142 diverse populations. Nature *538*, 201–206.

S16. Kılınç, G.M., Omrak, A., Özer, F., Günther, T., Büyükkarakaya, A.M., Bıçakçı, E., Baird, D., Dönertaş, H.M., Ghalichi, A., Yaka, R., et al. (2016). The Demographic Development of the First Farmers in Anatolia. Curr. Biol. *26*, 2659–2666.

S17. Fu, Q., Posth, C., Hajdinjak, M., Petr, M., Mallick, S., Fernandes, D., Furtwängler, A., Haak, W., Meyer, M., Mittnik, A., et al. (2016). The genetic history of Ice Age Europe. Nature *534*, 200–205.

S18. Cassidy, L.M., Martiniano, R., Murphy, E.M., Teasdale, M.D., Mallory, J., Hartwell, B., and Bradley, D.G. (2016). Neolithic and Bronze Age migration to Ireland and establishment of the insular Atlantic genome. Proc. Natl. Acad. Sci. U. S. A. *113*, 368–373.

S19. Broushaki, F., Thomas, M.G., Link, V., López, S., van Dorp, L., Kirsanow, K., Hofmanová, Z., Diekmann, Y., Cassidy, L.M., Díez-Del-Molino, D., et al.

(2016). Early Neolithic genomes from the eastern Fertile Crescent. Science *353*, 499–503.

S20. González-Fortes, G., Jones, E.R., Lightfoot, E., Bonsall, C., Lazar, C., Grandal-d'Anglade, A., Garralda, M.D., Drak, L., Siska, V., Simalcsik, A., et al. (2017). Paleogenomic Evidence for Multi-generational Mixing between Neolithic Farmers and Mesolithic Hunter-Gatherers in the Lower Danube Basin. Curr. Biol. *27*, 1801–1810.e10.

S21. Jones, E.R., Zarina, G., Moiseyev, V., Lightfoot, E., Nigst, P.R., Manica, A., Pinhasi, R., and Bradley, D.G. (2017). The Neolithic Transition in the Baltic Was Not Driven by Admixture with Early European Farmers. Curr. Biol. *27*, 576–582.

S22. Lipson, M., Szécsényi-Nagy, A., Mallick, S., Pósa, A., Stégmár, B., Keerl, V., Rohland, N., Stewardson, K., Ferry, M., Michel, M., et al. (2017). Parallel palaeogenomic transects reveal complex genetic history of early European farmers. Nature *551*, 368–372.

S23. Martiniano, R., Cassidy, L.M., Ó'Maoldúin, R., McLaughlin, R., Silva, N.M., Manco, L., Fidalgo, D., Pereira, T., Coelho, M.J., Serra, M., et al. (2017). The population genomics of archaeological transition in west Iberia: Investigation of ancient substructure using imputation and haplotype-based methods. PLoS Genet. *13*, e1006852.

S24. Sikora, M., Seguin-Orlando, A., Sousa, V.C., Albrechtsen, A., Korneliussen, T., Ko, A., Rasmussen, S., Dupanloup, I., Nigst, P.R., Bosch, M.D., et al. (2017). Ancient genomes show social and reproductive behavior of early Upper Paleolithic foragers. Science *358*, 659–662.

S25. Mathieson, I., Alpaslan-Roodenberg, S., Posth, C., Szécsényi-Nagy, A., Rohland, N., Mallick, S., Olalde, I., Broomandkhoshbacht, N., Candilio, F., Cheronet, O., et al. (2018). The genomic history of southeastern Europe. Nature *555*, 197–203.

S26. Fregel, R., Méndez, F.L., Bokbot, Y., Martín-Socas, D., Camalich-Massieu, M.D., Santana, J., Morales, J., Ávila-Arcos, M.C., Underhill, P.A., Shapiro, B., et al. (2018). Ancient genomes from North Africa evidence prehistoric migrations to the Maghreb from both the Levant and Europe. Proc. Natl. Acad. Sci. U. S. A. *115*, 6774–6779.

S27. Olalde, I., Brace, S., Allentoft, M.E., Armit, I., Kristiansen, K., Booth, T., Rohland, N., Mallick, S., Szécsényi-Nagy, A., Mittnik, A., et al. (2018). The Beaker phenomenon and the genomic transformation of northwest Europe. Nature *555*, 190–196.

S28. Günther, T., Malmström, H., Svensson, E.M., Omrak, A., Sánchez-Quinto, F., Kılınç, G.M., Krzewińska, M., Eriksson, G., Fraser, M., Edlund, H., et al. (2018). Population genomics of Mesolithic Scandinavia: Investigating early

postglacial migration routes and high-latitude adaptation. PLoS Biol. *16*, e2003703.

S29. Harney, É., May, H., Shalem, D., Rohland, N., Mallick, S., Lazaridis, I., Sarig, R., Stewardson, K., Nordenfelt, S., Patterson, N., et al. (2018). Ancient DNA from Chalcolithic Israel reveals the role of population mixture in cultural transformation. Nat. Commun. *9*, 3336.

S30. de Barros Damgaard, P., Martiniano, R., Kamm, J., Víctor Moreno-Mayar, J., Kroonen, G., Peyrot, M., Barjamovic, G., Rasmussen, S., Zacho, C., Baimukhanov, N., et al. (2018). The first horse herders and the impact of early Bronze Age steppe

S31. Valdiosera, C., Günther, T., Vera-Rodríguez, J.C., Ureña, I., Iriarte, E., Rodríguez-Varela, R., Simões, L.G., Martínez-Sánchez, R.M., Svensson, E.M., Malmström, H., et al. (2018). Four millennia of Iberian biomolecular prehistory illustrate the impact of prehistoric migrations at the far end of Eurasia. Proc. Natl. Acad. Sci. U. S. A. *115*, 3428–3433.

S32. Brace, S., Diekmann, Y., Booth, T.J., van Dorp, L., Faltyskova, Z., Rohland, N., Mallick, S., Olalde, I., Ferry, M., Michel, M., et al. (2019). Ancient genomes indicate population replacement in Early Neolithic Britain. Nat Ecol Evol *3*, 765–771.

S33. González-Fortes, G., Tassi, F., Trucchi, E., Henneberger, K., Paijmans, J.L.A., Díez-Del-Molino, D., Schroeder, H., Susca, R.R., Barroso-Ruíz, C., Bermudez, F.J., et al. (2019). A western route of prehistoric human migration from Africa into the Iberian Peninsula. Proc. Biol. Sci. *286*, 20182288.

S34. Olalde, I., Mallick, S., Patterson, N., Rohland, N., Villalba-Mouco, V., Silva, M., Dulias, K., Edwards, C.J., Gandini, F., Pala, M., et al. (2019). The genomic history of the Iberian Peninsula over the past 8000 years. Science *363*, 1230–1234.

S35. Narasimhan, V.M., Patterson, N., Moorjani, P., Rohland, N., Bernardos, R., Mallick, S., Lazaridis, I., Nakatsuka, N., Olalde, I., Lipson, M., et al. (2019). The formation of human populations in South and Central Asia. Science *365*.

S36. Feldman, M., Fernández-Domínguez, E., Reynolds, L., Baird, D., Pearson, J., Hershkovitz, I., May, H., Goring-Morris, N., Benz, M., Gresky, J., et al. (2019). Late Pleistocene human genome suggests a local origin for the first farmers of central Anatolia. Nat. Commun. *10*, 1218.

S37. Sánchez-Quinto, F., Malmström, H., Fraser, M., Girdland-Flink, L., Svensson, E.M., Simões, L.G., George, R., Hollfelder, N., Burenhult, G., Noble, G., et al. (2019). Megalithic tombs in western and northern Neolithic Europe were linked to a kindred society. Proc. Natl. Acad. Sci. U.

S. A. *116*, 9469–9474.

S38. Scheib, C.L., Hui, R., D’Atanasio, E., Wohns, A.W., Inskip, S.A., Rose, A., Cessford, C., O’Connell, T.C., Robb, J.E., Evans, C., et al. (2019). East Anglian early Neolithic monument burial linked to contemporary Megaliths. Ann. Hum. Biol. *46*, 145–149.

S39. Antonio, M.L., Gao, Z., Moots, H.M., Lucci, M., Candilio, F., Sawyer, S., Oberreiter, V., Calderon, D., Devitofranceschi, K., Aikens, R.C., et al. (2019). Ancient Rome: A genetic crossroads of Europe and the Mediterranean. Science *366*, 708–714.

S40. Cassidy, L.M., Maoldúin, R.Ó., Kador, T., Lynch, A., Jones, C., Woodman, P.C., Murphy, E., Ramsey, G., Dowd, M., Noonan, A., et al. (2020). A dynastic elite in monumental Neolithic society. Nature *582*, 384–388.

S41. Fernandes, D.M., Mittnik, A., Olalde, I., Lazaridis, I., Cheronet, O., Rohland, N., Mallick, S., Bernardos, R., Broomandkhoshbacht, N., Carlsson, J., et al. (2020). The spread of steppe and Iranian-related ancestry in the islands of the western Mediterranean. Nature Ecology & Evolution *4*, 334–345.

S42. Rivollat, M., Jeong, C., Schiffels, S., Küçükkalıpçı, İ., Pemonge, M.-H., Rohrlach, A.B., Alt, K.W., Binder, D., Friederich, S., Ghesquière, E., et al. (2020). Ancient genome-wide DNA from France highlights the complexity of interactions between Mesolithic hunter-gatherers and Neolithic farmers. Sci Adv *6*, eaaz5344.

S43. Ruden, D., Cingolani, P., Patel, V., Coon, M., Nguyen, T., Land, S., and Lu, X. (2012). Using Drosophila melanogaster as a Model for Genotoxic Chemical Mutational Studies with a New Program, SnpSift. Front. Genet. *3*.
