# Supplementary material for: Ancient Maltese genomes and the genetic geography of Neolithic Europe
Source: Curr Biol. 2022 Jun 20;32(12):2668–2680.e6. doi: 10.1016/j.cub.2022.04.069 (PMC9245899; doi:10.1016/j.cub.2022.04.069)
Supplement: Document S2. Article plus supplemental information [file mmc4.pdf]

Current Biology

Article

Ancient Maltese genomes and the genetic geography of Neolithic Europe

Graphical abstract

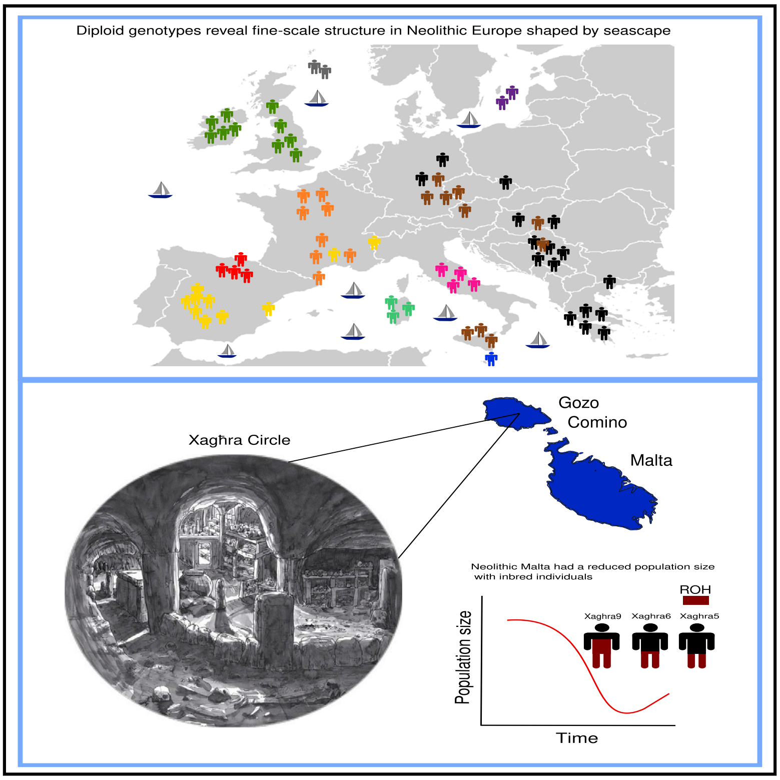

Highlights

- Three inbred genomes from Malta, dated around 2500 BC
- As in moderns, the genetic structure of Neolithic genomes is shaped by geography
- Genomic insularity of island Neolithic populations
- A marked distinction between the Danubian and Mediterranean farming expansion routes

Authors

Bruno Ariano, Valeria Mattiangeli, Emily M. Breslin, ..., Shyam Gopalakrishnan, Lara M. Cassidy, Daniel G. Bradley

Correspondence

fck245@ku.dk (S.G.),
cassidsl1@tcd.ie (L.M.C.),
dbradley@tcd.ie (D.G.B.)

In brief

Using high-coverage genomes from ancient Maltese, Ariano et al. show that they had signals of both recent and more distant inbreeding, indicating small population size. Using diploid genome-wide data, the authors explore the genetic geography of Neolithic Europe which, echoing modern patterns, is shaped by physical topography, especially its seascapes.

Ariano et al., 2022, Current Biology *32*, 2668–2680
June 20, 2022 © 2022 The Authors. Published by Elsevier Inc.
https://doi.org/10.1016/j.cub.2022.04.069

CellPress

CellPress
OPEN ACCESS

Current Biology

Article

Ancient Maltese genomes and the genetic geography of Neolithic Europe

Bruno Ariano,[1] Valeria Mattiangeli,[1] Emily M. Breslin,[1] Eóin W. Parkinson,[2] T. Rowan McLaughlin,[3] Jess E. Thompson,[4] Ronika K. Power,[5] Jay T. Stock,[6] Bernardette Mercieca-Spiteri,[7] Simon Stoddart,[4] Caroline Malone,[8] Shyam Gopalakrishnan,[9,*] Lara M. Cassidy,[1,*] and Daniel G. Bradley[1,10,*]
[1]Smurfit Institute of Genetics, Trinity College Dublin, Dublin 2, Ireland
[2]Department of Classics and Archaeology, University of Malta, Msida 2080, Malta
[3]Department of Scientific Research, The British Museum, Great Russell Street, London WC1B 3DG, UK
[4]McDonald Institute for Archaeological Research, University of Cambridge, Downing Street, Cambridge CB2 3EJ, UK
[5]Department of History and Archaeology, Macquarie University, 25B Wally's Walk, Sydney, NSW, Australia
[6]Department of Anthropology, Western University, 1151 Richmond St, London, ON N6G 2V4, Canada
[7]Superintendence of Cultural Heritage, St Christopher Street, Valletta 2000, Malta
[8]School of Natural and Built Environment, Queen's University Belfast, Elmwood Avenue, Belfast, UK
[9]GLOBE Institute, University of Copenhagen, Øster Farimagsgade 5, 1353 København K, Denmark
[10]Lead contact
*Correspondence: fck245@ku.dk (S.G.), cassidsl1@tcd.ie (L.M.C.), dbradley@tcd.ie (D.G.B.)
https://doi.org/10.1016/j.cub.2022.04.069

SUMMARY

Archaeological consideration of maritime connectivity has ranged from a biogeographical perspective that considers the sea as a barrier to a view of seaways as ancient highways that facilitate exchange. Our results illustrate the former. We report three Late Neolithic human genomes from the Mediterranean island of Malta that are markedly enriched for runs of homozygosity, indicating inbreeding in their ancestry and an effective population size of only hundreds, a striking illustration of maritime isolation in this agricultural society. In the Late Neolithic, communities across mainland Europe experienced a resurgence of hunter-gatherer ancestry, pointing toward the persistence of different ancestral strands that subsequently admixed. This is absent in the Maltese genomes, giving a further indication of their genomic insularity. Imputation of genome-wide genotypes in our new and 258 published ancient individuals allowed shared identity-by-descent segment analysis, giving a fine-grained genetic geography of Neolithic Europe. This highlights the differentiating effects of seafaring Mediterranean expansion and also island colonization, including that of Ireland, Britain, and Orkney. These maritime effects contrast profoundly with a lack of migratory barriers in the establishment of Central European farming populations from Anatolia and the Balkans.

INTRODUCTION

The importance of sea travel in prehistory is clear from the rapid westward spread of agriculture from its origins in the Near East along the Mediterranean littoral, including its very early appearance in Cyprus circa (c.) 10,600 years ago.[1] However, the consideration of seascapes in prehistory has varied, with a biogeographical view emphasizing the sea as a barrier and, alternately, a view that posits seaways as efficient corridors of connectivity.[2,3] Ancient genomics has confirmed the demic, or migratory, nature of Neolithic expansion but has also given some illustrations of retardation of seaborne genetic exchange. For example, the Sardinian Bronze Age population was unaffected by an influx of Steppe ancestry that changed the genomes of contemporaneous mainland Europeans,[4,5] and Irish Mesolithic genomes show the signatures of small population size, which were absent in corresponding continental hunter gatherers (HGs).[6]

The first settlements in the Maltese islands were Neolithic, dated from the sixth millennium BC. These developed through a series of cultural phases, with some material indications of external connectivity,[7] but faded from 3600 BC when pottery and architecture started to show distinctive features.[8] One example was the development of multi-chambered rock-cut tombs, such as that at Xagħra circle, Gozo (Figure 1).[7] This monumentalized underground tomb yielded the remains of hundreds of individuals[9] and underwent remodeling and enlargement until around 2500 BC when it was abandoned, possibly as part of a wider population decline or replacement.

To examine the demography of Late Neolithic Malta, we sequenced genomes from Xagħra circle. The elucidation of fine structure among closely related groups such as European Neolithic populations is challenging and requires the resolution afforded by genealogical methods.[10] Therefore, to examine these in a wider context, we additionally imputed genome-wide diploid genotypes from published ancient genomes and assessed haplotype sharing within and between genomes to estimate genetic geography and demographies across Neolithic Europe.

 © 2022 The Authors. Published by Elsevier Inc.
This is an open access article under the CC BY license (http://creativecommons.org/licenses/by/4.0/).

CellPress
OPEN ACCESS

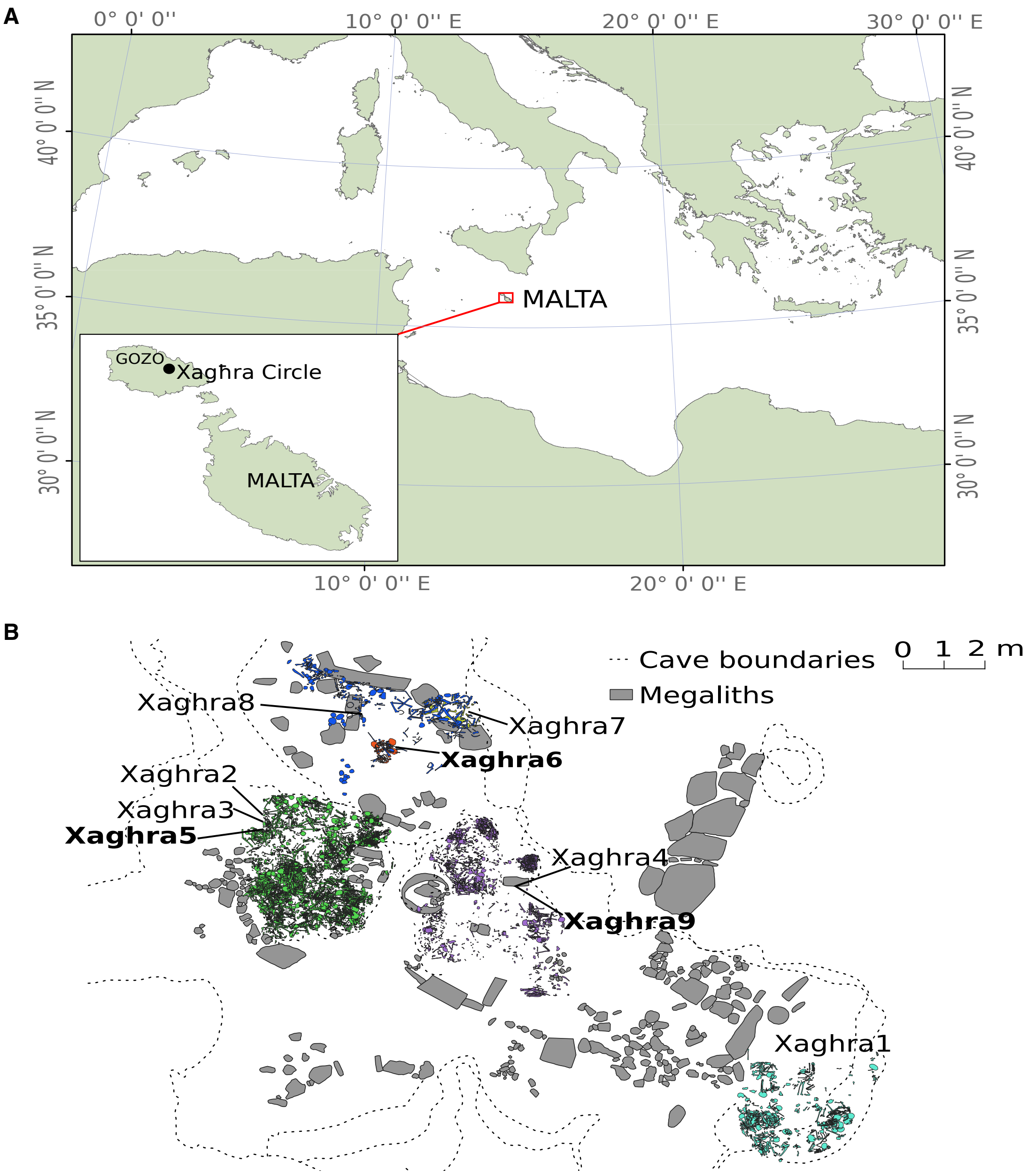

Figure 1. Location of the samples within the Maltese Xagħra Circle site

(A) Location of the Maltese archipelago within southern Europe.

(B) Plan of the Xagħra Circle site showing skeletal remains from the archaeological contexts studied. Colors represent different archaeological layers (green, 783; blue, 951; lilac, 960; yellow, 111; turquoise, 1,241; orange, 1,307).

RESULTS AND DISCUSSION

Genomes from a south Mediterranean island

The retrieval of ancient genomes from warm climates is highly challenging, and the island of Gozo in the Maltese archipelago is one of the southernmost contexts in Europe (Figure 1). However, from nine human petrous bone and tooth samples from the Late Neolithic Xagħra Circle excavation, three yielded excellent endogenous DNA content (13%, 17%, and 39%; Table 1). This likely reflects enhanced preservation within this underground limestone cave burial complex (hypogeum), and these samples, Xaghra5, 6, and 9, were shotgun sequenced to an average genome-wide coverage of 1.24×, 0.98×, and 7.52×, respectively.

Malta was one of the final regions of Europe to be inhabited, with little evidence of human presence prior to the arrival of Neolithic communities, which were established on the archipelago by 5500 cal. BC.[11] These were associated with a developed style of impressed pottery (Għar Dalam ware) that represented a regional variant of Sicilian and southwestern Italian ceramics.

Table 1. Summary of samples from Late Neolithic contexts at the Xagħra circle

Sample ID	Date BC	Genomic sex	Endogenous DNA (%)	Genome coverage	mtDNA HG	Y-chr HG	X-chr contamination estimate (%)	mtDNA contamination estimate (%)
Xaghra1	2575–2520	Female	1.9	0.05	–	–	–	–
Xaghra2	2550–2350	Unknown	0.06	<0.01	–	–	–	–
Xaghra3	2550–2350	Male	0.42	<0.01	–	–	–	–
Xaghra4	2535–2475	Female	1.7	0.03	–	–	–	–
Xaghra5	2550–2350	Male	37	1.24	K1a	H2	0.6 (0.27)	0.533 (0.14)
Xaghra6	2900–2750	Female	12	0.98	V	–	–	0.787 (0.11)
Xaghra7	2875–2615	Female	0.16	<0.01	–	–	–	–
Xaghra8	2575–2470	Female	0.03	<0.01	–	–	–	–
Xaghra9	2530–2400	Male	15	7.52	H4a1	G2a2a1a3	1.1 (0.33)	0.340 (0.13)

Date ranges have been estimated using the 95% CI of Bayesian chronological models.[7] Uniparental haplogroups and mtDNA contamination estimates were reported in Ariano et al.[11] 95% confidence intervals are reported in parenthesis for contamination levels. See also STAR Methods.

Accordingly, we find the genomes from Xagħra Circle share highest levels of drift with the Early Neolithic populations of Italy and Greece, followed by Middle Neolithic and Chalcolithic populations from Italy and Sicily, as estimated using outgroup f_3-statistics (Data S2F; Figure S2). Levels of Western HG (WHG) admixture have been shown to vary across European Neolithic samples,[12–17] particularly through time. To examine levels of WHG ancestry within our Neolithic sample, we applied the qpAdm method to each site, binning genomes into 500-year intervals. We observe WHG ancestral components to increase significantly with time (Figure S3; $r^2 = -0.52$, p value = 2.8e−4). Interestingly, the Xagħra Circle site shows the lowest amount of HG ancestry (6.8% ± 2.5%) among other groups from the Later Neolithic (Figure S3). This may reflect a shielding by its island context from the dissemination of admixtures with persisting WHG populations that widely influenced mainland populations and which have been estimated to occur as late as 3800 BC.[17] This resonates with observations from Sardinian populations, which show a constant degree of WHG ancestry stretching through the Neolithic to Bronze Age periods.[4,5] Using *D*-statistics, we also tested for gene flow related to North African, Caucasus HG, Neolithic Iranian farmer, and Yamnaya-steppe groups into the Maltese populations, to the exclusion of the Greek and Italian Early Neolithic. We obtained no statistical evidence for admixture (Data S2).

The imputation of diploid genotypes from low coverage shotgun sequence data has been successfully utilized for the characterization of fine-scale structure and patterns of inbreeding in ancient populations.[6,18,19] We applied an imputation pipeline (STAR Methods) to the Maltese samples using Impute2,[20,21] as well as to 117 individuals for which sufficient shotgun sequence (>0.4×) was available (Data S1A). The resulting diploid genotypes were merged with relevant ancient Italian samples from a published imputed dataset.[22] We also extended an imputation pipeline to individuals that had been sampled with a SNP capture protocol[13,14,23] using Beagle v.4.1,[24,25] achieving an accuracy in the predictions of heterozygous genotypes of 95% (Figure S6). After excluding 4 samples with high numbers of missing genotypes, this gave a final comparative dataset of 271 Neolithic and 86 HG ancient individuals from western Eurasia[5,6,12,13,15,16,18,19,22,26–51] (Data S1A). Comparisons of runs of homozygosity (ROHs) estimates, using diploid high-coverage data and the alternate imputation pipelines for individuals with both shotgun sequence and SNP capture data, show very high concordance ($r^2 = 0.99$; Figure S4) and validate the combined analysis of this dataset. Comparisons of identity-by-descent (IBD) scores between the data types also show no evidence of bias (Figure S4).

Xagħra circle genomes show outlying homozygosity levels and a historically restricted population size

Genome-wide diploid data allow haplotype-based assessments of population diversity—specifically, the distribution of shared ancestry within genomes, using ROH, and the distribution between individuals by identifying shared tracts that are identical by descent. ROH analysis shows outlying behavior by the Maltese genomes. Xaghra9 has the second most extreme levels of long ROH (>5 cM) yet reported in prehistory: an assertion secured by its high genome coverage (Figure S5) and a confirmatory analysis using a second-analysis method (using ROHan[52]), which estimated 19.12% of the genome under ROH. This is only exceeded within an individual deposited in an Irish passage tomb (NG10, Newgrange10), who was the offspring of a first-order consanguineous union.[6] However, Xaghra9 has a ROH size spectrum that has less skew toward very long tracts of identity (>15 cM; Figure 2A).

CellPress
OPEN ACCESS

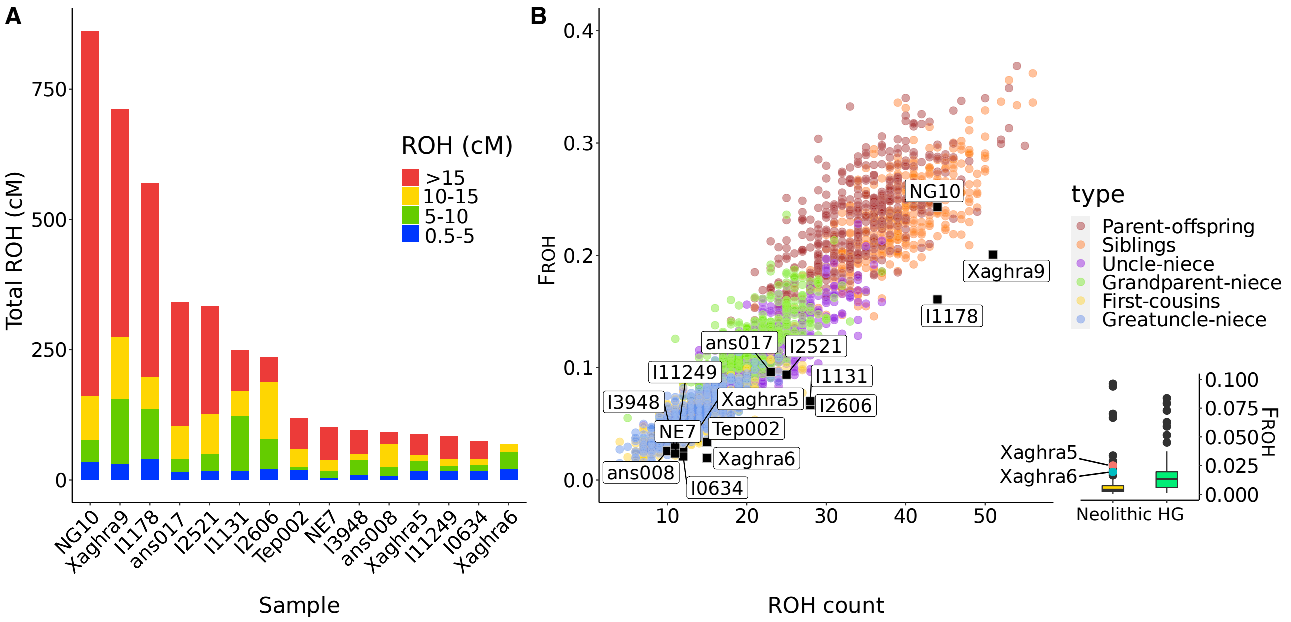

Figure 2. ROH and inbreeding coefficient (F_{ROH}) distributions among ancient Neolithic populations

(A) Runs of homozygosity totals for Maltese samples are within the upper extreme in the Neolithic distribution. Xaghra9 particularly has a very high total and includes long runs indicating familial inbreeding—however, not as pronounced as Newgrange10 (NG10).

See also Figure S5 and Data S1D.

(B) Simulations of ROH spectra using specific genealogical scenarios (n = 400 for each) generate parameter distributions consistent with individuals from Gotland, Copper Age Israel, and Newgrange, Ireland having resulted from recent familial inbreeding via simple pedigree loops. However, both the Xaghra9 and Israeli Copper Age (I1178) individuals have different spectra; higher contributions from short ROH indicate that they likely have multiple, complex inbreeding loops in their ancestry. The inset compares boxplots of ancient European hunter-gatherer (HG) and Neolithic F_{ROH} values; the Xaghra5 and Xaghra6 genomes are more typical of the former, despite having material culture of the latter.

To explore this signal, a range of consanguineous parentages were simulated, and the number of ROH segments with the total fraction of the genome in these ROH (F_{ROH}) were plotted and compared with ancient individuals (Figure 2B). Unlike NG10, Xaghra9 falls at the edge of the distribution seen for matings between first-degree relatives and may result from a more complex combination of multiple inbreeding loops within his genealogy. However, this is similar to Israeli Chalcolithic sample I1178[43] (F_{ROH} = 0.16; Data S1D), who was previously identified in a different analysis as a possible product of brother-sister or parent-offspring consanguinity.[53] Consequently, we do not assert a precise scenario for the parentage of Xaghra9. To focus on very recent inbreeding, we repeated this analysis twice, considering only ROH segments longer than 10 cM and then longer than 15 cM; for each, Xaghra9 remains at the edge or outside the sibling mating cluster. Given the small size and relative isolation of Gozo island, it is possible that the inbreeding loops that gave rise to the Xaghra9 genome are the result of both recent genealogical inbreeding and a historically small ancestral population size. This interpretation is supported by the observation of less pronounced but relatively inflated levels of the fraction of the genome in ROH in the other two Maltese genomes (Xaghra5, Xaghra6; Figures 2A, 2B, and S5A), one of which predates Xaghra9 by ~400 years. The values for these two samples are more typical of those found in European HGs, who maintained smaller population sizes than later farming populations (Figure 2B). To investigate further, we used levels of ROH within a range of 4–20 cM and a maximum likelihood framework[53,54] to estimate effective population size, giving a total of 515 (95% confidence interval [CI] 397–633) individuals.

We also calculated effective population size for the Xagħra population using the software IBDNe,[55] which leverages patterns of IBD sharing between individuals. For comparison, we included other European Neolithic sites with more than 90 IBD segments shared between individuals in total. Xagħra, and to a lesser extent the remains from the Tomb of the Eagles on Isbister in the Orkney islands, show recent dips in population size, with the Late Neolithic Maltese sample giving a 30-generation average of only 382 individuals (Figure 3A).

Thus, these preserved Maltese samples show a genomic signature of an unusually small and restricted population, a signal which is distributed over a period of at least 400 years. Interestingly, the two later individuals (Xaghra5 and Xaghra9) derive from a turning point in Maltese prehistory c. 2450 BC, with a reducing density of radiocarbon dates[56] and marked worsening in diet and nutritional status.[57] A long-term trend toward increasing aridity and thinning soils that began as early as 5500 BC[58] seems to be driving these changes, implying the Late Neolithic population was less than the Early Neolithic carrying capacity estimate of two or three thousand individuals for Gozo island (67 sq km).[58] This is only a small multiple of our calculated effective population size values, which are therefore

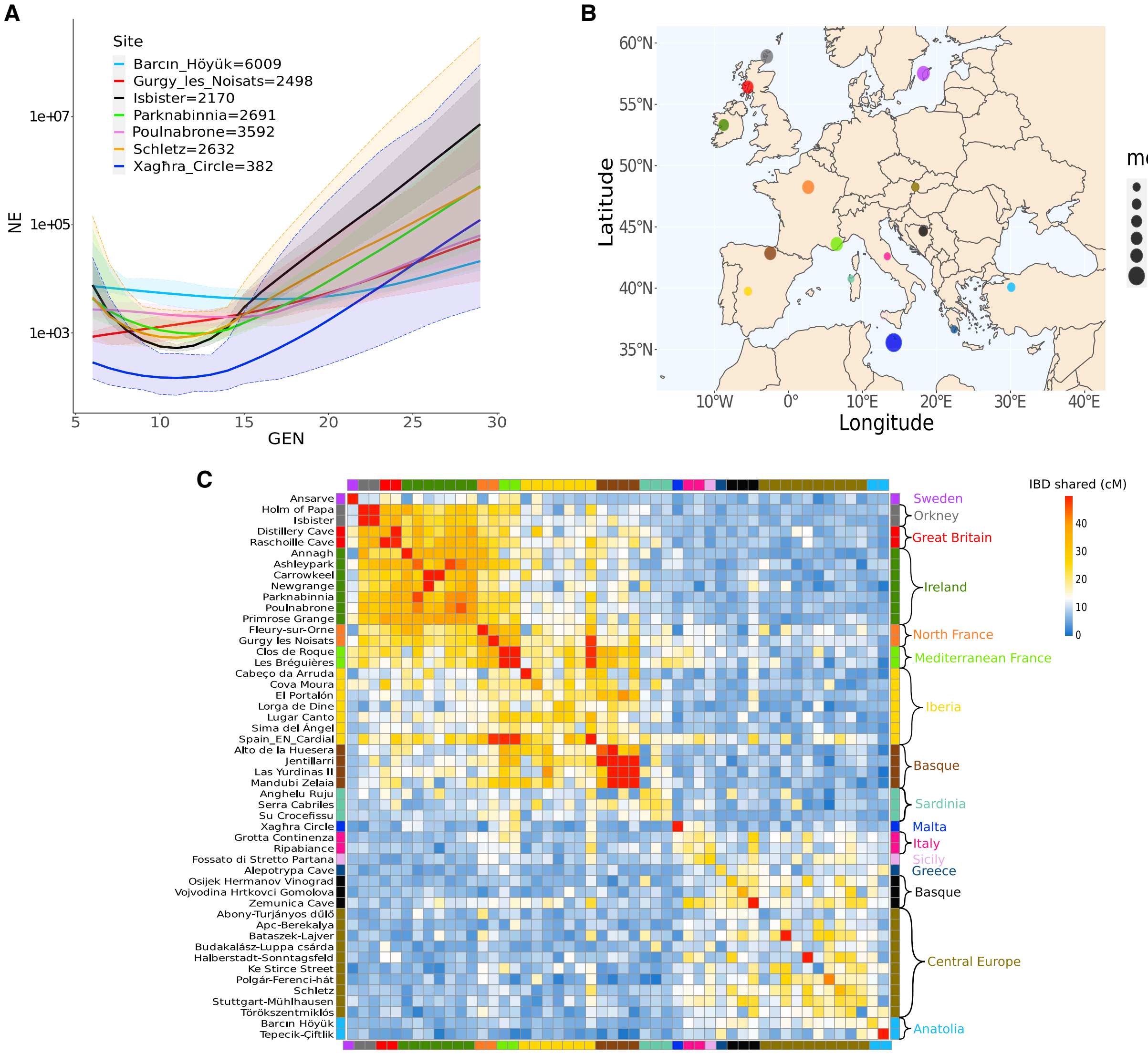

Figure 3. IBD within and between sites

(A) Population size estimated for site samples showing at least 90 IBD shared segments. The Xagħra Circle plot estimates a marked size reduction in recent ancestry and has the lowest 30-generation average effective population size of 382. 95% confidence intervals are represented in shade colors.

(B) Average IBD length in cM shared within groups defined in (C). Malta, Gotland, and the Scottish islands display the highest within-site IBD average values, suggesting ancestral population restriction.

(C) IBD sharing heatmap among those sites with two or more representatives. Note a British/Irish cluster in the top left. French individuals share some affinity with this but also cluster with Iberians and Sardinians in a large west Mediterranean group. Two island samples, Xagħra (Malta) and Ansarve (Gotland), are relatively distinct, and all other sites show a loose affinity in an East Mediterranean/Central European grouping.

See also Data S1B.

not surprising. However, these estimates suggest isolation, with mating networks largely confined within the island's shores. Several strands of evidence suggest our sample is representative of the wider Neolithic community on Gozo. First, the age profile of Xagħra burials coincides closely with expectations of the mortality rates of a full early farming community, namely high infant and adolescent mortality and a relatively equal balance of adult males and females.[59] Second, the spatial analysis of the mortuary remains suggests a rich and elaborate treatment of the burials as one community,[9,60,61]-12166603600450-2819404725035 and finally, the chosen samples are drawn from different parts of the site and span the entirety of its use.

Archaeological evidence for overseas communication with Malta in this period is mixed. Some products, such as obsidian, types of chert, and polished stone were definitely imported.[60,62] However, these tend to be small, of high prestige value, and have a finished state when they appear, suggesting they may not have been accompanied by a substantial volume of human traffic. Moreover, the means of cultivation of crops, raising of animals, and construction were local in nature, consistent with a degree of insularity.

CellPress
OPEN ACCESS

Body mass index analysis of Neolithic populations

The carving and circulation of apparently obese human figurines was a marked feature of the late Maltese Neolithic,[8] perhaps mirroring an unusual genetic predisposition within a restricted gene pool. Accordingly, we performed a polygenic risk score analysis on body mass index using the summary statistics from the UK Biobank dataset, but found that the three Maltese Neolithic individuals sampled do not give atypical risk values compared with other Neolithic individuals (Figure S8).

Haplotype sharing within and between Neolithic sites suggests restricted island population sizes and seaborne founder effects

Shared IBD is sensitive to recent common ancestry and, because it is a genealogical rather than a frequency-based method,[63,64] it may be less skewed by factors such as the differences in levels of HG ancestry that are known among European Neolithic populations.[12–16] Figure 3C (Data S1B) shows a heatmap of the average IBD length ($\geq$2 cM) observed between and within European Neolithic archaeological sites with more than one imputed genome, after filtering for related individuals. We observe the highest within-site values for samples from small islands, with Xagħra (Malta) producing the most extreme result, followed by Ansarve (Gotland), Holm of Papa (Orkney), and Isbister (Orkney), supporting restricted population histories for insular Neolithic societies. Figure 3B plots the averaged values for different geographical regions and reveals an additional trend of higher within-group IBD sharing in the north and west of the continent relative to the south and east.

This geographical difference also manifests in patterns of between-site sharing (Figure 3C), with three distinct regional clusters apparent. The Basque region, situated between the Atlantic Ocean and the western Pyrenees mountains, shows extremely inflated values between Later Neolithic sites, implying a degree of geographic isolation. Close genealogical ties are also seen across Britain and Ireland, consistent with a seaborne colonization of the islands derived from a single or closely related founder populations. Finally, we observe French sites clustering together, within which extreme sharing is observed between two Early Neolithic sites from Southern France, potentially reflective of the enclave colonization process that characterized Neolithic expansion across the Mediterranean. To explore this signal further, we considered three sites from the earliest horizon of the Spanish Neolithic (c. 5500–5000 BC), previously excluded given only a single sample was available from each. Surprisingly, despite the large geographic distances between them (Data S1A), these three individuals show very high levels of sharing with one another and with the Mediterranean French sites, despite large differences in their HG ancestral contribution (Figure S3). This implies a population size restriction accompanied Neolithic migration into the Western Mediterranean.

Neolithic genes mirror geography

To explore the potential impact of maritime colonization and continental topography on Neolithic genetic structure, we carried out principal component analysis (PCA) on a matrix of pairwise IBD sharing between individual imputed ancient individuals (Figure 4), as well as ChromoPainter and clustering using fineSTRUCTURE analysis[65] (Figure 5). In addition to these haplotype-based methods, we also applied an allele frequency-based approach (EEMS, estimated effective migration surface[66]).

Results from each show a convergence on the existence of three clusters: first, the Western Mediterranean, including Iberian, French, and Sardinian individuals; second, the Eastern Mediterranean, featuring Greek, Balkan, and Anatolian individuals as well as Central Europeans; and third, the British and Irish archipelago. These are visible as blocks in the IBD heatmap of Figure 3C and form three apices of variation in the PCA (Figure 4). They also form separate primary branches in a fineSTRUCTURE tree (Figure 5).[65] Intermediate samples are also intermediate in geography. For example, in the PCA plot, which visibly mirrors geography (Figure 4), Northern French samples are placed close to Iberians but also stretch toward the British and Irish cluster. Also, the mid-Mediterranean samples from Sardinia, Malta, Sicily and Italy fall between the western and eastern poles.

Neolithic populations migrated through Europe via two major routes, an overland transfer into Central Europe and a maritime dissemination along the Mediterranean coast.[67] The most striking feature in our analyses are the contrasting outcomes of these two processes. Particularly, there is minimal distinction between central European individuals and their source populations in the Balkans and Anatolia, whereas the separation of western European individuals from those in the southeast forms the primary divide in the data.

This supports a model of agricultural expansion into Central Europe from the Balkans that involved substantial numbers of migrants and strong backward communication during the dissemination of the Linearbandkeramik (LBK) complex, with populations remaining relatively well connected throughout the Neolithic period.

To explore further, we also EEMSs using a stepping-stone model and a distance matrix computed from allele frequencies[66]; Figure 6 shows cold- and hotspots of estimated migration rates within Neolithic Europe. The communication corridor between Anatolia, the Balkans, and Central Europe is the most striking feature of this analysis and contrasts strongly with east-west barriers in the Mediterranean sea, the Alpine region, and further north where the two Neolithic migratory streams are purported to meet.[51] In common with the other approaches, EEMS does not take account of temporal differences among samples, which would be expected to be a differentiating factor. For example, the barrier between English samples and the continent might be less pronounced with the addition of more contemporaneous French genomes. However, we assert that the major divisions are explained at least partially by geography. These correspond with those that emerge in the haplotype-informed fineSTRUCTURE analysis, where sample dates are also plotted (Figure 5). From this it is clear that genomes separate into different groups despite overlapping contemporaneity across the basal branches. Also, there are considerable temporal differences within clusters, particularly among the samples in the Anatolian-Central European high communication corridor.

The rapid Neolithic colonization of the Western Mediterranean from the east was associated with the impressed cardial complex and likely took place through iterative coastal placements along the northern maritime littoral.[68,69] Models of this process based on archaeological data indicate that long-range voyaging

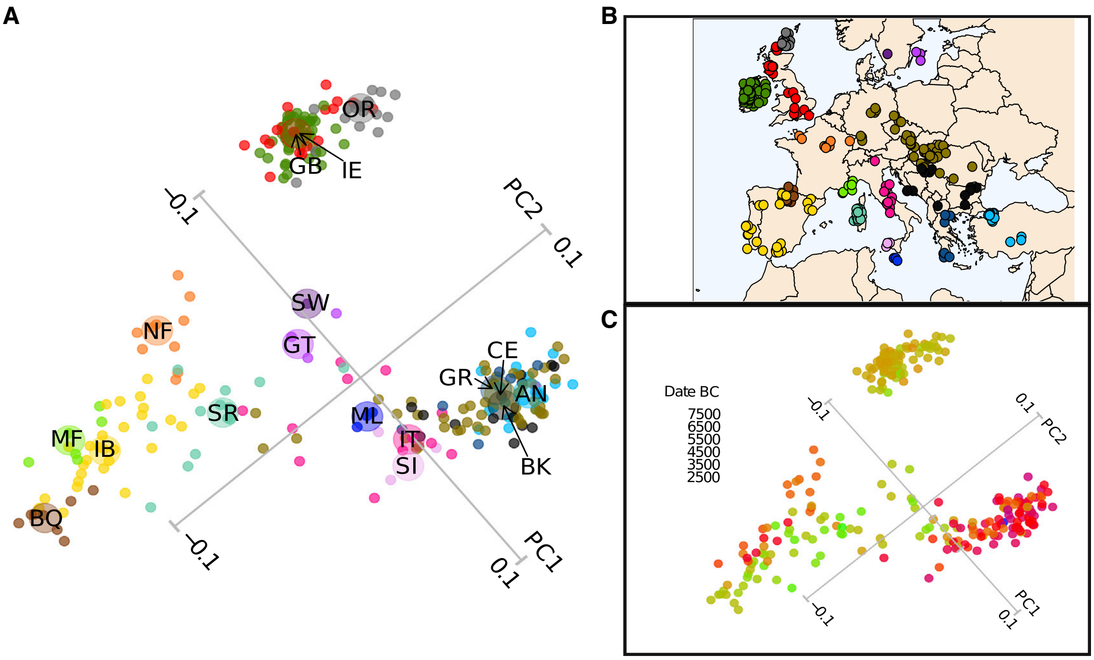

Figure 4. Principal components analysis of shared IBD

(A) Principal components analysis of European Neolithic imputed ancient individuals based on total length of identity-by-descent segments. The variance explained by PC1 and PC2 are, respectively, 19% and 4.7%. Regional origins of samples are denoted by color, and two letter codes in the inset map and centroids for each group are denoted as larger circles in the plot. Three main clusters emerge: Britain/Ireland, France/Iberia, and Anatolia/Balkans/Central Europe. Island Mediterranean Maltese, Sardinian, and Sicilian samples, along with Italian individuals, fall between the latter two groups in approximate geographical sequence. Orcadian samples also distinguish from the broader British/Irish group, as do Basque sites within Iberia. AN, Anatolia; BK, Balkans; BQ, Basque; CE, Central Europe; GB, Great Britain; GR, Greece; GT, Gotland island; IB, Iberia; IE, Ireland; IT, Italy; MF, Mediterranean France; ML, Malta; NF, Northern France; OR, Orkney; SI, Sicily; SR, Sardinia; SW, Sweden mainland; and B, location of each sample colored using the PCA as reference.
See also Data S1B.
(B) Geographic location of the samples shown in (A).
(C) Same principal component plot as (A), with samples colored according to their estimated age in years BC.
See also Data S1B.

is required to explain the speed of agricultural spread, which was significantly faster than that seen in Central Europe.[67,70] Our results accord with a limited capacity of sea craft used in this cabotage, which likely restricted pioneer numbers and subsequent backward exchange. We infer that the observed east-west genomic distinction derives at least partially from this foundational process, as earlier individuals plot toward the extremes in the PCA graph with mid and Late Neolithic individuals showing a more central tendency (Figure 4C). The sharp divide between eastern and western Europe echoes the analysis of French and neighboring Neolithic genomes by Rivollat et al.,[51] who also identify that the two Neolithic streams differed in their degree of ancestral admixture with European HGs. However, this difference in ancestry is less marked in comparison with earlier western genomes, for example, those of the Iberian Early Neolithic (Figure S3).

British and Irish populations form a sister grouping to the Mediterranean Neolithic in the second fineSTRUCTURE branching (Figure 5) and visibly show IBD affinity (Figure 3C), according with prior assertions that they primarily owe their origins to this southern migratory stream.[6,15,41,49] However, their maritime separation is mirrored by a degree of cluster distinction (Figures 3 and 4) and an estimated migration barrier (Figure 6). Interestingly, Irish and mainland British individuals do not separate from each other as clusters in any of our analyses, supporting shared elements of a rapid foundation process c. 3800 BC.[71] This is an additional indication of the absence of significant batch effects, as the British were imputed from SNP capture data and the Irish from shotgun-sequenced libraries. However, fineSTRUCTURE confirms the emerging distinctiveness of (SNP-captured) Orcadian individuals, as well as that of Basque Late Neolithic sites (Figure 5), also captured in patterns of IBD sharing (Figures 3C and 4). An additional marker of separation is that Orkney islander ancient genomes have also recently been found to show unusual majority retention of male lineages across the Neolithic-Bronze Age transition,[72] a feature unique within Northern and Central Europe.

Conclusions

Basque, Orcadian, and Irish distinctiveness emerged in pioneering studies of modern human genetic variation,[73–76] and genome-scale investigation has compellingly recapitulated the geography of Europe in PCA, particularly its maritime features.[77] It is striking that these same features emerge independently within data from an earlier genomic era in the same continent, speaking to the repeated shaping of genetic variation by the same physical topography, particularly its seascapes. One of the great debates of prehistory is the level of maritime

CellPress
OPEN ACCESS

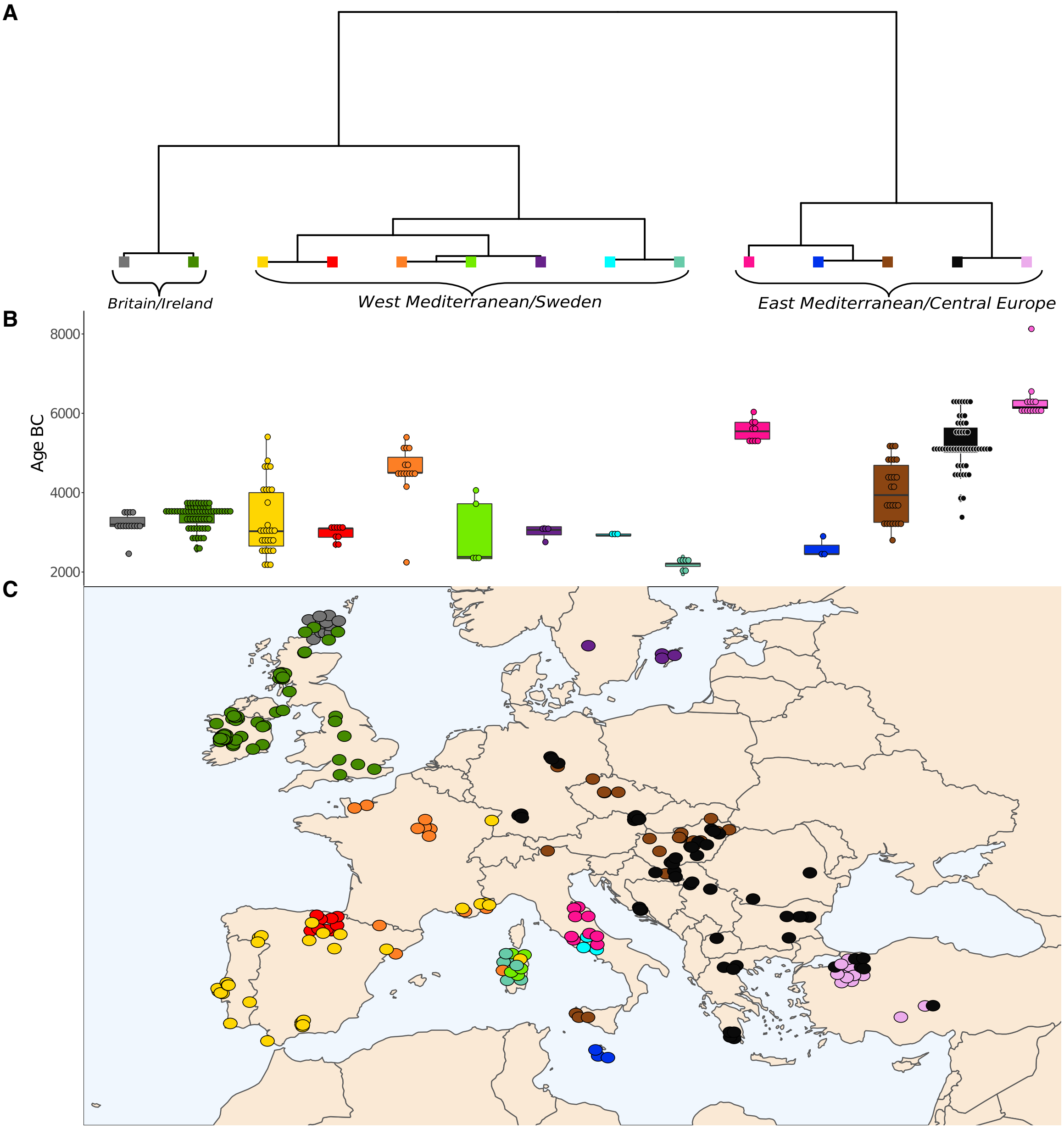

Figure 5. Fine population structure analysis of European Neolithic populations

(A) fineSTRUCTURE tree of Neolithic European populations. From left to right, three main branches define, respectively, Britain and Ireland, West Mediterranean, and East Mediterranean as higher order population groupings. The Maltese samples emerge as a cluster and group with Italian and late Central European Neolithic groups.

(B) Boxplot indicating the age in years BC of each group defined by fineSTRUCTURE. Note the structuring of the East Mediterranean/Central Europe grouping by both age and geography.

(C) Location of samples colored according to their groups defined by fineSTRUCTURE. A jitter of 0.6 was used to visualize points.

See also Data S1C.

connectivity during the course of millennia and how that connectivity interacted with marine technology and cultural response.[2] We suggest that relationships among ancient European populations indicate that sea travel was one driver of genomic differentiation during the establishment of the Neolithic. On a wide scale, multiple analyses highlight the genetic separation between Western Mediterranean sites and their source Eastern Mediterranean populations. This resulted from coastal seaborne colonization and contrasts sharply with the lack of differentiation associated with the overland establishment of Central European LBK populations from southeastern Europe and Anatolia. That maritime routes are a retardant rather than accelerant of genetic exchange is also clear from small islands. Orcadian, Gotland, and Maltese genomes show signals of high ROH or within-site

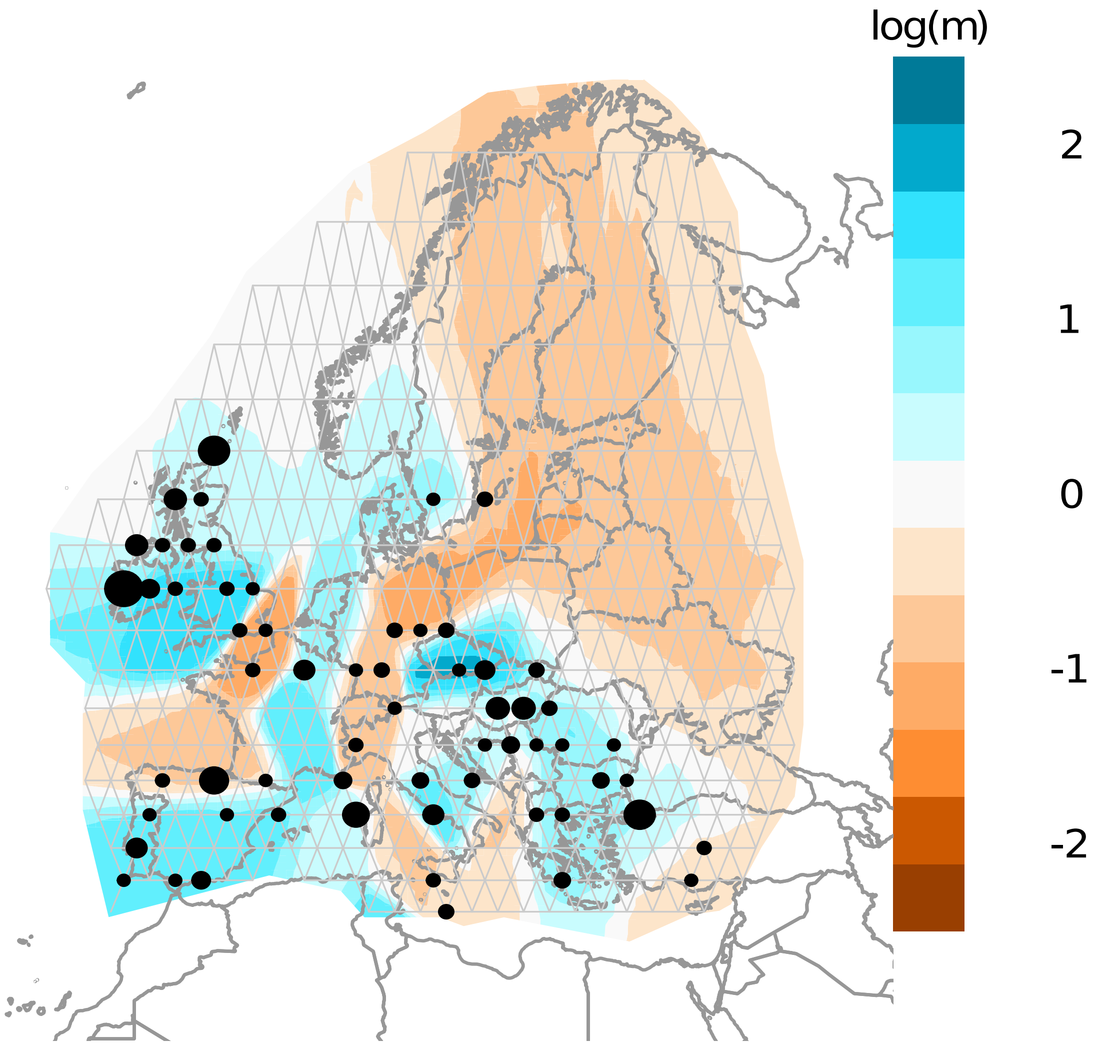

Figure 6. Estimated Neolithic effective migration surface (EEMS)
Computed using a stepping-stone model and imputed allele frequency data, migration rates are plotted as $\log_{10}$ of the mean effective migration rate. Blue regions are surfaces over which genomic similarity is implied, orange denote barriers to genetic exchange. Dots represent the location of the samples in the constructed grid, while their size indicates the number of samples. Apparent barriers separate Western and Eastern Europe and mainland Europe from Britain and Ireland.

IBD, suggesting limited populations. Particularly, effective population size estimates of only several hundred for the Late Neolithic Maltese Xagħra site suggest a population with mating networks no larger than the island of Gozo and are a powerful example of genomic insularity in prehistory.

STAR★METHODS

Detailed methods are provided in the online version of this paper and include the following:

- KEY RESOURCES TABLE
- RESOURCE AVAILABILITY
  - Lead contact
  - Materials availability
  - Data and code availability
- EXPERIMENTAL MODEL AND SUBJECT DETAILS
  - Xagħra (Brochtorff) circle
- METHOD DETAILS
  - Sampling and DNA extraction
  - Radiocarbon analysis
  - Library preparation
  - DNA sequencing
- QUANTIFICATION AND STATISTICAL ANALYSIS
  - Reads processing
  - Contamination estimation and sex determination
  - Population structure analysis
  - *F*-statistics
  - Genotype imputation
  - ROH and inbreeding analysis

- Pedigree simulation
- IBD analysis
- Population size estimates
- Chromopainter/fineSTRUCTURE
- Estimated effective migration surface
- BMI analysis

SUPPLEMENTAL INFORMATION

Supplemental information can be found online at https://doi.org/10.1016/j.cub.2022.04.069.

ACKNOWLEDGMENTS

This ancient genomic work was funded by the Science Foundation Ireland/Health Research Board/Wellcome Trust Biomedical Research Partnership Investigator award no. 205072 to D.G.B., "Ancient Genomics and the Atlantic Burden." The authors would also like to thank the DJEI/DES/SFI/HEA Irish Centre for High-End Computing (ICHEC) for their support and for providing the computational resources. The human remains investigated for this analysis were studied as part of the European Research Council advanced grant 323727 "FRAGSUS" Project, PI Caroline Malone. Permission to study the remains was kindly granted by Sharon Sultana, curator at the National Museum of Archaeology, Valletta, and authorized by the Superintendence of Cultural Heritage (Malta). We thank the Cambridge Metabolic network for organizing a stimulating conference on obesity, which led to analysis of the genetic predisposition toward obesity in the Neolithic populations of Europe (BMI). We also would like to extend our gratitude to Harald Ringbauer and two anonymous reviewers for insightful suggestions.

AUTHOR CONTRIBUTIONS

Conceptualization, D.G.B., B.A., S.S., and C.M.; investigation, B.A., L.M.C., E.M.B., and D.G.B.; (aDNA), V.M. and B.A.; formal analysis, B.A., E.M.B., S.G., and L.M.C.; writing – original draft, B.A., D.G.B., and L.M.C.; writing – review & editing, all authors; funding acquisition, D.G.B. and C.M.; supervision, D.G.B., L.M.C., and V.M.

DECLARATION OF INTERESTS

The authors declare no competing interests.

INCLUSION AND DIVERSITY

We worked to ensure diversity in experimental samples through the selection of the genomic datasets. One or more of the authors of this paper self-identifies as a member of the LGBTQ+ community. One or more of the authors of this paper self-identifies as living with a disability. One or more of the authors of this paper received support from a program designed to increase minority representation in science. While citing references scientifically relevant for this work, we also actively worked to promote gender balance in our reference list.

Received: November 4, 2021
Revised: February 7, 2022
Accepted: April 22, 2022
Published: May 18, 2022

REFERENCES

1. Vigne, J.-D., Briois, F., Zazzo, A., Willcox, G., Cucchi, T., Thiébault, S., Carrère, I., Franel, Y., Touquet, R., Martin, C., et al. (2012). First wave of cultivators spread to Cyprus at least 10,600 y ago. Proc. Natl. Acad. Sci. USA *109*, 8445–8449.
2. Rainbird, P. (2007). The Archaeology of Islands (Cambridge University Press).
3. Cherry, J.F., and Leppard, T.P. (2014). A little history of Mediterranean island prehistory. In The Cambridge Prehistory of the Bronze and Iron Age Mediterranean (Cambridge University Press), pp. 10–24.
4. Marcus, J.H., Posth, C., Ringbauer, H., Lai, L., Skeates, R., Sidore, C., Beckett, J., Furtwängler, A., Olivieri, A., Chiang, C.W.K., et al. (2020). Genetic history from the Middle Neolithic to present on the Mediterranean island of Sardinia. Nat. Commun. *11*, 939.
5. Fernandes, D.M., Mittnik, A., Olalde, I., Lazaridis, I., Cheronet, O., Rohland, N., Mallick, S., Bernardos, R., Broomandkhoshbacht, N., Carlsson, J., et al. (2020). The spread of steppe and Iranian-related ancestry in the islands of the western Mediterranean. Nat. Ecol. Evol. *4*, 334–345.
6. Cassidy, L.M., Maoldúin, R.Ó., Kador, T., Lynch, A., Jones, C., Woodman, P.C., Murphy, E., Ramsey, G., Dowd, M., Noonan, A., et al. (2020). A dynastic elite in monumental Neolithic society. Nature *582*, 384–388.
7. Malone, C., Cutajar, N., McLaughlin, T.R., Mercieca-Spiteri, B., Pace, A., Power, R.K., Stoddart, S., Sultana, S., Ramsey, C.B., Dunbar, E., et al. (2019). Island questions: the chronology of the Brochtorff Circle at Xagħra, Gozo, and its significance for the Neolithic sequence on Malta. Archaeol. Anthropol. Sci. *11*, 4251–4306.
8. Malone, C., and Stoddart, S. (2016). Figurines of Malta. In The Oxford Handbook of Prehistoric Figurines (Oxford University Press).
9. Thompson, J.E., Parkinson, E.W., McLaughlin, T.R., Barratt, R.P., Power, R.K., Mercieca-Spiteri, B., Stoddart, S., and Malone, C. (2020). Placing and remembering the dead in late Neolithic Malta: bioarchaeological and spatial analysis of the Xagħra Circle Hypogeum, Gozo. World Archaeol. *52*, 71–89.
10. Ralph, P., and Coop, G. (2013). The geography of recent genetic ancestry across Europe. PLoS Biol. *11*, e1001555.
11. Ariano, B., Mattiangeli, V., Cassidy, L., McLaughlin, R., Power, R., Stock, J., Mercieca-Spiteri, B., Stoddart, S., Malone, C., and Daniel, G. (2022). Bradley aDNA Origins of the early Maltese people. In Temple People: Bioarchaeology, Resilience and Culture in Prehistoric Malta, S. Stoddart, R.K. Power, J.E. Thompson, B. Mercieca-Spiteri, T.R. McLaughlin, E.W. Parkinson, A. Pace, and C. Malone, eds. (McDonald Institute for Archaeological Research Cambridge).
12. Gamba, C., Jones, E.R., Teasdale, M.D., McLaughlin, R.L., Gonzalez-Fortes, G., Mattiangeli, V., Domboróczki, L., Kővári, I., Pap, I., Anders, A., et al. (2014). Genome flux and stasis in a five millennium transect of European prehistory. Nat. Commun. *5*, 5257.
13. Mathieson, I., Lazaridis, I., Rohland, N., Mallick, S., Patterson, N., Roodenberg, S.A., Harney, E., Stewardson, K., Fernandes, D., Novak, M., et al. (2015). Genome-wide patterns of selection in 230 ancient Eurasians. Nature *528*, 499–503.
14. Haak, W., Lazaridis, I., Patterson, N., Rohland, N., Mallick, S., Llamas, B., Brandt, G., Nordenfelt, S., Harney, E., Stewardson, K., et al. (2015). Massive migration from the steppe was a source for Indo-European languages in Europe. Nature *522*, 207–211.
15. Cassidy, L.M., Martiniano, R., Murphy, E.M., Teasdale, M.D., Mallory, J., Hartwell, B., and Bradley, D.G. (2016). Neolithic and Bronze Age migration to Ireland and establishment of the insular Atlantic genome. Proc. Natl. Acad. Sci. USA *113*, 368–373.
16. Skoglund, P., Malmström, H., Omrak, A., Raghavan, M., Valdiosera, C., Günther, T., Hall, P., Tambets, K., Parik, J., Sjögren, K.-G., et al. (2014). Genomic diversity and admixture differs for Stone-Age Scandinavian foragers and farmers. Science *344*, 747–750.
17. Seguin-Orlando, A., Donat, R., Der Sarkissian, C., Southon, J., Thèves, C., Manen, C., Tchérémissinoff, Y., Crubézy, E., Shapiro, B., Deleuze, J.-F., et al. (2021). Heterogeneous Hunter-Gatherer and Steppe-Related Ancestries in Late Neolithic and Bell Beaker genomes from present-day France. Curr. Biol. *31*, 1072–1083.e10.
18. Jones, E.R., Gonzalez-Fortes, G., Connell, S., Siska, V., Eriksson, A., Martiniano, R., McLaughlin, R.L., Gallego Llorente, M., Cassidy, L.M.,

Gamba, C., et al. (2015). Upper Palaeolithic genomes reveal deep roots of modern Eurasians. Nat. Commun. *6*, 8912.

19. Martiniano, R., Cassidy, L.M., Ó'Maoldúin, R., McLaughlin, R., Silva, N.M., Manco, L., Fidalgo, D., Pereira, T., Coelho, M.J., Serra, M., et al. (2017). The population genomics of archaeological transition in west Iberia: investigation of ancient substructure using imputation and haplotype-based methods. PLoS Genet. *13*, e1006852.

20. Howie, B.N., Donnelly, P., and Marchini, J. (2009). A flexible and accurate genotype imputation method for the next generation of genome-wide association studies. PLoS Genet. *5*, e1000529.

21. Howie, B., Marchini, J., and Stephens, M. (2011). Genotype imputation with thousands of genomes. G3 (Bethesda) *1*, 457–470.

22. Antonio, M.L., Gao, Z., Moots, H.M., Lucci, M., Candilio, F., Sawyer, S., Oberreiter, V., Calderon, D., Devitofranceschi, K., Aikens, R.C., et al. (2019). Ancient Rome: a genetic crossroads of Europe and the Mediterranean. Science *366*, 708–714.

23. Fu, Q., Hajdinjak, M., Moldovan, O.T., Constantin, S., Mallick, S., Skoglund, P., Patterson, N., Rohland, N., Lazaridis, I., Nickel, B., et al. (2015). An early modern human from Romania with a recent Neanderthal ancestor. Nature *524*, 216–219.

24. Browning, S.R., and Browning, B.L. (2007). Rapid and accurate haplotype phasing and missing-data inference for whole-genome association studies by use of localized haplotype clustering. Am. J. Hum. Genet. *81*, 1084–1097.

25. Browning, B.L., and Browning, S.R. (2016). Genotype imputation with millions of reference samples. Am. J. Hum. Genet. *98*, 116–126.

26. Keller, A., Graefen, A., Ball, M., Matzas, M., Boisguerin, V., Maixner, F., Leidinger, P., Backes, C., Khairat, R., Forster, M., et al. (2012). New insights into the Tyrolean Iceman's origin and phenotype as inferred by whole-genome sequencing. Nat. Commun. *3*, 698.

27. Lazaridis, I., Patterson, N., Mittnik, A., Renaud, G., Mallick, S., Kirsanow, K., Sudmant, P.H., Schraiber, J.G., Castellano, S., Lipson, M., et al. (2014). Ancient human genomes suggest three ancestral populations for present-day Europeans. Nature *513*, 409–413.

28. Seguin-Orlando, A., Korneliussen, T.S., Sikora, M., Malaspinas, A.-S., Manica, A., Moltke, I., Albrechtsen, A., Ko, A., Margaryan, A., Moiseyev, V., et al. (2014). Paleogenomics. Genomic structure in Europeans dating back at least 36,200 years. Science *346*, 1113–1118.

29. Olalde, I., Allentoft, M.E., Sánchez-Quinto, F., Santpere, G., Chiang, C.W.K., DeGiorgio, M., Prado-Martinez, J., Rodríguez, J.A., Rasmussen, S., Quilez, J., et al. (2014). Derived immune and ancestral pigmentation alleles in a 7,000-year-old Mesolithic European. Nature *507*, 225–228.

30. Olalde, I., Schroeder, H., Sandoval-Velasco, M., Vinner, L., Lobón, I., Ramirez, O., Civit, S., García Borja, P., Salazar-García, D.C., Talamo, S., et al. (2015). A common genetic origin for early farmers from Mediterranean cardial and Central European LBK cultures. Mol. Biol. Evol. *32*, 3132–3142.

31. Günther, T., Valdiosera, C., Malmström, H., Ureña, I., Rodriguez-Varela, R., Sverrisdóttir, Ó.O., Daskalaki, E.A., Skoglund, P., Naidoo, T., Svensson, E.M., et al. (2015). Ancient genomes link early farmers from Atapuerca in Spain to modern-day Basques. Proc. Natl. Acad. Sci. USA *112*, 11917–11922.

32. Allentoft, M.E., Sikora, M., Sjögren, K.-G., Rasmussen, S., Rasmussen, M., Stenderup, J., Damgaard, P.B., Schroeder, H., Ahlström, T., Vinner, L., et al. (2015). Population genomics of Bronze Age Eurasia. Nature *522*, 167–172.

33. Hofmanová, Z., Kreutzer, S., Hellenthal, G., Sell, C., Diekmann, Y., Díez-Del-Molino, D., van Dorp, L., López, S., Kousathanas, A., Link, V., et al. (2016). Early farmers from across Europe directly descended from Neolithic Aegeans. Proc. Natl. Acad. Sci. USA *113*, 6886–6891.

34. Kılınç, G.M., Omrak, A., Özer, F., Günther, T., Büyükkarakaya, A.M., Bıçakçı, E., Baird, D., Dönertaş, H.M., Ghalichi, A., Yaka, R., et al. (2016). The demographic development of the first farmers in Anatolia. Curr. Biol. *26*, 2659–2666.

35. Fu, Q., Posth, C., Hajdinjak, M., Petr, M., Mallick, S., Fernandes, D., Furtwängler, A., Haak, W., Meyer, M., Mittnik, A., et al. (2016). The genetic history of Ice Age Europe. Nature *534*, 200–205.

36. González-Fortes, G., Jones, E.R., Lightfoot, E., Bonsall, C., Lazar, C., Grandal-d'Anglade, A., Garralda, M.D., Drak, L., Siska, V., Simalcsik, A., et al. (2017). Paleogenomic evidence for multi-generational mixing between Neolithic Farmers and Mesolithic Hunter-Gatherers in the Lower Danube Basin. Curr. Biol. *27*, 1801–1810.e10.

37. Jones, E.R., Zarina, G., Moiseyev, V., Lightfoot, E., Nigst, P.R., Manica, A., Pinhasi, R., and Bradley, D.G. (2017). The Neolithic transition in the Baltic was not driven by admixture with early European farmers. Curr. Biol. *27*, 576–582.

38. Lipson, M., Szécsényi-Nagy, A., Mallick, S., Pósa, A., Stégmár, B., Keerl, V., Rohland, N., Stewardson, K., Ferry, M., Michel, M., et al. (2017). Parallel palaeogenomic transects reveal complex genetic history of early European farmers. Nature *551*, 368–372.

39. Sikora, M., Seguin-Orlando, A., Sousa, V.C., Albrechtsen, A., Korneliussen, T., Ko, A., Rasmussen, S., Dupanloup, I., Nigst, P.R., Bosch, M.D., et al. (2017). Ancient genomes show social and reproductive behavior of early Upper Paleolithic foragers. Science *358*, 659–662.

40. Mathieson, I., Alpaslan-Roodenberg, S., Posth, C., Szécsényi-Nagy, A., Rohland, N., Mallick, S., Olalde, I., Broomandkhoshbacht, N., Candilio, F., Cheronet, O., et al. (2018). The genomic history of southeastern Europe. Nature *555*, 197–203.

41. Olalde, I., Brace, S., Allentoft, M.E., Armit, I., Kristiansen, K., Booth, T., Rohland, N., Mallick, S., Szécsényi-Nagy, A., Mittnik, A., et al. (2018). The Beaker phenomenon and the genomic transformation of northwest Europe. Nature *555*, 190–196.

42. Günther, T., Malmström, H., Svensson, E.M., Omrak, A., Sánchez-Quinto, F., Kılınç, G.M., Krzewińska, M., Eriksson, G., Fraser, M., Edlund, H., et al. (2018). Population genomics of Mesolithic Scandinavia: investigating early postglacial migration routes and high-latitude adaptation. PLoS Biol. *16*, e2003703.

43. Harney, É., May, H., Shalem, D., Rohland, N., Mallick, S., Lazaridis, I., Sarig, R., Stewardson, K., Nordenfelt, S., Patterson, N., et al. (2018). Ancient DNA from Chalcolithic Israel reveals the role of population mixture in cultural transformation. Nat. Commun. *9*, 3336.

44. Valdiosera, C., Günther, T., Vera-Rodríguez, J.C., Ureña, I., Iriarte, E., Rodríguez-Varela, R., Simões, L.G., Martínez-Sánchez, R.M., Svensson, E.M., Malmström, H., et al. (2018). Four millennia of Iberian biomolecular prehistory illustrate the impact of prehistoric migrations at the far end of Eurasia. Proc. Natl. Acad. Sci. USA *115*, 3428–3433.

45. Brace, S., Diekmann, Y., Booth, T.J., van Dorp, L., Faltyskova, Z., Rohland, N., Mallick, S., Olalde, I., Ferry, M., Michel, M., et al. (2019). Ancient genomes indicate population replacement in Early Neolithic Britain. Nat. Ecol. Evol. *3*, 765–771.

46. González-Fortes, G., Tassi, F., Trucchi, E., Henneberger, K., Paijmans, J.L.A., Díez-Del-Molino, D., Schroeder, H., Susca, R.R., Barroso-Ruíz, C., Bermudez, F.J., et al. (2019). A western route of prehistoric human migration from Africa into the Iberian Peninsula. Proc. Biol. Sci. *286*, 20182288.

47. Olalde, I., Mallick, S., Patterson, N., Rohland, N., Villalba-Mouco, V., Silva, M., Dulias, K., Edwards, C.J., Gandini, F., Pala, M., et al. (2019). The genomic history of the Iberian Peninsula over the past 8000 years. Science *363*, 1230–1234.

48. Narasimhan, V.M., Patterson, N., Moorjani, P., Rohland, N., Bernardos, R., Mallick, S., Lazaridis, I., Nakatsuka, N., Olalde, I., Lipson, M., et al. (2019). The formation of human populations in South and Central Asia. Science *365*.

49. Sánchez-Quinto, F., Malmström, H., Fraser, M., Girdland-Flink, L., Svensson, E.M., Simões, L.G., George, R., Hollfelder, N., Burenhult, G., Noble, G., et al. (2019). Megalithic tombs in western and northern

CellPress
OPEN ACCESS

Neolithic Europe were linked to a kindred society. Proc. Natl. Acad. Sci. USA *116*, 9469–9474.

50. Scheib, C.L., Hui, R., D'Atanasio, E., Wohns, A.W., Inskip, S.A., Rose, A., Cessford, C., O'Connell, T.C., Robb, J.E., Evans, C., et al. (2019). East Anglian early Neolithic monument burial linked to contemporary Megaliths. Ann. Hum. Biol. *46*, 145–149.

51. Rivollat, M., Jeong, C., Schiffels, S., Küçükkalıpçı, İ., Pemonge, M.-H., Rohrlach, A.B., Alt, K.W., Binder, D., Friederich, S., Ghesquière, E., et al. (2020). Ancient genome-wide DNA from France highlights the complexity of interactions between Mesolithic hunter-gatherers and Neolithic farmers. Sci. Adv. *6*, eaaz5344.

52. Renaud, G., Hanghøj, K., Korneliussen, T.S., Willerslev, E., and Orlando, L. (2019). Joint estimates of heterozygosity and runs of homozygosity for modern and ancient samples. Genetics *212*, 587–614.

53. Ringbauer, H., Novembre, J., and Steinrücken, M. (2021). Parental relatedness through time revealed by runs of homozygosity in ancient DNA. Nat. Commun. *12*, 5425.

54. Fernandes, D.M., Sirak, K.A., Ringbauer, H., Sedig, J., Rohland, N., Cheronet, O., Mah, M., Mallick, S., Olalde, I., Culleton, B.J., et al. (2021). A genetic history of the pre-contact Caribbean. Nature *590*, 103–110.

55. Browning, S.R., and Browning, B.L. (2015). Accurate non-parametric estimation of recent effective population size from segments of identity by descent. Am. J. Hum. Genet. *97*, 404–418.

56. McLaughlin, R., Parkinson, E.W., Reimer, P.J., and Malone, C. (2020). Dating Maltese prehistory. In Temple places: Excavating cultural sustainability in prehistoric Malta (McDonald Institute for Archaeological Research).

57. Stoddart, S., Power, R.K., Thompson, J.E., Mercieca-Spiteri, B., Rowan McLaughlin, T., Parkinson, E.W., Pace, A., and Malone, C. (2022). Temple People: Bioarchaeology, Resilience and Culture in Prehistoric Malta (McDonald Institute for Archaeological Research).

58. French, C., Hunt, C.O., Grima, R., McLaughlin, R., Stoddart, S., and Malone, C. (2020). Temple Landscapes: Fragility, Change and Resilience of Holocene Environments in the Maltese Islands (Mcdonald Institute for Archaeological Research).

59. Stoddart, S., Malone, C., Mason, S., Trump, B., and Trump, D.H. (2009). The Tarxien Phase Levels: Spatial and Stratigraphic Analysis and Reconstruction (McDonald Institute for Archaeological Research).

60. Malone, C., and Stoddart, S. (2009). Mortuary Customs in Prehistoric Malta: Excavations at the Brochtorff Circle at Xagħra (1987–94) (McDonald Institute for Archeological Research).

61. Malone, C., McLaughlin, R., Mercieca-Spiteri, B., Parkinson, E., Power, R., Stock, J., Stoddart, S., and Thompson, J. (2018). The Social Implications of Death in Prehistoric Malta. In Gathered in Death: Archaeological and Ethnological Perspectives on Collective Burial and Social Organisation (Presses Universitaires de Louvain).

62. Malone, C., Grima, R., McLaughlin, R., Parkinson, E.W., Stoddart, S., and Vella, N. (2020). Temple Places: Excavating Cultural Sustainability in Prehistoric Malta (University of Cambridge Repository).

63. Thompson, E.A. (2013). Identity by descent: variation in meiosis, across genomes, and in populations. Genetics *194*, 301–326.

64. Mooney, J.A., Huber, C.D., Service, S., Sul, J.H., Marsden, C.D., Zhang, Z., Sabatti, C., Ruiz-Linares, A., Bedoya, G., et al.; Costa Rica/Colombia Consortium for Genetic Investigation of Bipolar Endophenotypes (2018). Understanding the hidden complexity of Latin American population isolates. Am. J. Hum. Genet. *103*, 707–726.

65. Lawson, D.J., Hellenthal, G., Myers, S., and Falush, D. (2012). Inference of population structure using dense haplotype data. PLoS Genet. *8*, e1002453.

66. Petkova, D., Novembre, J., and Stephens, M. (2016). Visualizing spatial population structure with estimated effective migration surfaces. Nat. Genet. *48*, 94–100.

67. Bocquet-Appel, J.-P., Naji, S., Vander Linden, M., and Kozlowski, J. (2012). Understanding the rates of expansion of the farming system in Europe. J. Archaeol. Sci. *39*, 531–546.

68. Zilhão, J. (2014). Early prehistoric navigation in the western Mediterranean: implications for the Neolithic transition in Iberia and the Maghreb. Eurasian Prehist. *11*, 185–200.

69. Cunliffe, S.B. (2017). On the Ocean: the Mediterranean and the Atlantic from Prehistory to AD 1500 (Oxford University Press).

70. Isern, N., Zilhão, J., Fort, J., and Ammerman, A.J. (2017). Modeling the role of voyaging in the coastal spread of the Early Neolithic in the West Mediterranean. Proc. Natl. Acad. Sci. USA *114*, 897–902.

71. Sheridan, A. (2010). The Neolithization of Britain and Ireland: the "Big Picture.". In Landscapes in Transition (Oxbow/Council for British Research in the Levant), pp. 89–105.

72. Dulias, K., Foodya, M.G.B., Justeaua, P., Silva, M., Martiniano, R., Oteo-García, G., Fichera, A., Rodrigues, S., Gandini, F., Meynert, A., et al. (2022). Ancient DNA at the Edge of the World: continental immigration and the persistence of Neolithic Male Lineages in Bronze Age Orkney. Proc. Natl. Acad Sci. USA *119*. e2108001119.

73. Cavalli-Sforza, L.L., Menozzi, P., and Piazza, A. (1994). The History and Geography of Human Genes (Princeton University Press).

74. Bodmer, W. (2015). Genetic characterization of human populations: From ABO to a genetic map of the British people. Genetics *199*, 267–279.

75. Leslie, S., Winney, B., Hellenthal, G., Davison, D., Boumertit, A., Day, T., Hutnik, K., Royrvik, E.C., Cunliffe, B., et al.; Wellcome Trust Case Control Consortium 2 (2015). The fine-scale genetic structure of the British population. Nature *519*, 309–314.

76. Mourant, A.E. (1947). The blood groups of the Basques. Nature *160*, 505.

77. Novembre, J., Johnson, T., Bryc, K., Kutalik, Z., Boyko, A.R., Auton, A., Indap, A., King, K.S., Bergmann, S., Nelson, M.R., et al. (2008). Genes mirror geography within Europe. Nature *456*, 98–101.

78. 1000 Genomes Project Consortium, Auton, A., Brooks, L.D., Durbin, R.M., Garrison, E.P., Kang, H.M., Korbel, J.O., Marchini, J.L., McCarthy, S., McVean, G.A., et al. (2015). A global reference for human genetic variation. Nature *526*, 68–74.

79. Martin, M. (2011). Cutadapt removes adapter sequences from high-throughput sequencing reads. EMBnet J. *17*, 10–12.

80. Schubert, M., Lindgreen, S., and Orlando, L. (2016). AdapterRemoval v2: rapid adapter trimming, identification, and read merging. BMC Res. Notes *9*, 88.

81. Li, H., and Durbin, R. (2009). Fast and accurate short read alignment with Burrows-Wheeler transform. Bioinformatics *25*, 1754–1760.

82. Li, H., Handsaker, B., Wysoker, A., Fennell, T., Ruan, J., Homer, N., Marth, G., Abecasis, G., and Durbin, R.; 1000 Genome Project Data Processing Subgroup (2009). The Sequence Alignment/Map format and SAMtools. Bioinformatics *25*, 2078–2079.

83. Okonechnikov, K., Conesa, A., and García-Alcalde, F. (2016). Qualimap 2: advanced multi-sample quality control for high-throughput sequencing data. Bioinformatics *32*, 292–294.

84. Skoglund, P., Storå, J., Götherström, A., and Jakobsson, M. (2013). Accurate sex identification of ancient human remains using DNA shotgun sequencing. J. Archaeol. Sci. *40*, 4477–4482.

85. McKenna, A., Hanna, M., Banks, E., Sivachenko, A., Cibulskis, K., Kernytsky, A., Garimella, K., Altshuler, D., Gabriel, S., Daly, M., et al. (2010). The Genome Analysis Toolkit: a MapReduce framework for analyzing next-generation DNA sequencing data. Genome Res. *20*, 1297–1303.

86. Chang, C.C., Chow, C.C., Tellier, L.C., Vattikuti, S., Purcell, S.M., and Lee, J.J. (2015). Second-generation PLINK: rising to the challenge of larger and richer datasets. GigaScience *4*, 7.

87. Patterson, N., Price, A.L., and Reich, D. (2006). Population structure and eigenanalysis. PLoS Genet. *2*, e190.

88. Patterson, N., Moorjani, P., Luo, Y., Mallick, S., Rohland, N., Zhan, Y., Genschoreck, T., Webster, T., and Reich, D. (2012). Ancient admixture in human history. Genetics *192*, 1065–1093.

89. Cingolani, P., Patel, V.M., Coon, M., Nguyen, T., Land, S.J., Ruden, D.M., et al. (2012). Using Drosophila melanogaster as a Model for Genotoxic Chemical Mutational Studies with a New Program, SnpSift. Front. Genet. *3*, 35.

90. Delaneau, O., Coulonges, C., and Zagury, J.-F. (2008). Shape-IT: new rapid and accurate algorithm for haplotype inference. BMC Bioinform. *9*, 540.

91. Caballero, M., Seidman, D.N., Qiao, Y., Sannerud, J., Dyer, T.D., Lehman, D.M., Curran, J.E., Duggirala, R., Blangero, J., Carmi, S., et al. (2019). Crossover interference and sex-specific genetic maps shape identical by descent sharing in close relatives. PLoS Genet. *15*, e1007979.

92. Campbell, C.L., Furlotte, N.A., Eriksson, N., Hinds, D., and Auton, A. (2015). Escape from crossover interference increases with maternal age. Nat. Commun. *6*, 6260.

93. Manichaikul, A., Mychaleckyj, J.C., Rich, S.S., Daly, K., Sale, M., and Chen, W.-M. (2010). Robust relationship inference in genome-wide association studies. Bioinformatics *26*, 2867–2873.

94. Browning, B.L., and Browning, S.R. (2013). Detecting identity by descent and estimating genotype error rates in sequence data. Am. J. Hum. Genet. *93*, 840–851.

95. Yang, D.Y., Eng, B., Waye, J.S., Dudar, J.C., and Saunders, S.R. (1998). Technical note: improved DNA extraction from ancient bones using silica-based spin columns. Am. J. Phys. Anthropol. *105*, 539–543.

96. MacHugh, D.E., Edwards, C.J., Bailey, J.F., Bancroft, D.R., and Bradley, D.G. (2000). The extraction and analysis of ancient DNA from bone and teeth: a survey of current methodologies. Anc. Biomol. *3*, 81–103.

97. Boessenkool, S., Hanghøj, K., Nistelberger, H.M., Der Sarkissian, C., Gondek, A.T., Orlando, L., Barrett, J.H., and Star, B. (2017). Combining bleach and mild predigestion improves ancient DNA recovery from bones. Mol. Ecol. Resour. *17*, 742–751.

98. Meyer, M., and Kircher, M. (2010). Illumina sequencing library preparation for highly multiplexed target capture and sequencing. Cold Spring Harb. Protoc. *2010*. pdb.prot5448.

99. Broushaki, F., Thomas, M.G., Link, V., López, S., van Dorp, L., Kirsanow, K., Hofmanová, Z., Diekmann, Y., Cassidy, L.M., Díez-Del-Molino, D., et al. (2016). Early Neolithic genomes from the eastern Fertile Crescent. Science *353*, 499–503.

100. Price, A.L., Patterson, N.J., Plenge, R.M., Weinblatt, M.E., Shadick, N.A., and Reich, D. (2006). Principal components analysis corrects for stratification in genome-wide association studies. Nat. Genet. *38*, 904–909.

101. Reich, D., Thangaraj, K., Patterson, N., Price, A.L., and Singh, L. (2009). Reconstructing Indian population history. Nature *461*, 489–494.

102. Mallick, S., Li, H., Lipson, M., Mathieson, I., Gymrek, M., Racimo, F., Zhao, M., Chennagiri, N., Nordenfelt, S., Tandon, A., et al. (2016). The Simons Genome Diversity Project: 300 genomes from 142 diverse populations. Nature *538*, 201–206.

103. Green, R.E., Krause, J., Briggs, A.W., Maricic, T., Stenzel, U., Kircher, M., Patterson, N., Li, H., Zhai, W., Fritz, M.H.-Y., et al. (2010). A draft sequence of the Neandertal genome. Science *328*, 710–722.

104. Fregel, R., Méndez, F.L., Bokbot, Y., Martín-Socas, D., Camalich-Massieu, M.D., Santana, J., Morales, J., Ávila-Arcos, M.C., Underhill, P.A., Shapiro, B., et al. (2018). Ancient genomes from North Africa evidence prehistoric migrations to the Maghreb from both the Levant and Europe. Proc. Natl. Acad. Sci. USA *115*, 6774–6779.

105. Gazal, S., Sahbatou, M., Perdry, H., Letort, S., Génin, E., and Leutenegger, A.-L. (2014). Inbreeding coefficient estimation with dense SNP data: comparison of strategies and application to HapMap III. Hum. Hered. *77*, 49–62.

106. McQuillan, R., Leutenegger, A.-L., Abdel-Rahman, R., Franklin, C.S., Pericic, M., Barac-Lauc, L., Smolej-Narancic, N., Janicijevic, B., Polasek, O., Tenesa, A., et al. (2008). Runs of homozygosity in European populations. Am. J. Hum. Genet. *83*, 359–372.

107. Bhérer, C., Campbell, C.L., and Auton, A. (2017). Refined genetic maps reveal sexual dimorphism in human meiotic recombination at multiple scales. Nat. Commun. *8*, 14994.

108. Schroeder, H., Margaryan, A., Szmyt, M., Theulot, B., Włodarczak, P., Rasmussen, S., Gopalakrishnan, S., Szczepanek, A., Konopka, T., Jensen, T.Z.T., et al. (2019). Unraveling ancestry, kinship, and violence in a Late Neolithic mass grave. Proc. Natl. Acad. Sci. USA *116*, 10705–10710.

109. Delaneau, O., Marchini, J., and Zagury, J.-F. (2011). A linear complexity phasing method for thousands of genomes. Nat. Methods *9*, 179–181.

CellPress
OPEN ACCESS

STAR★METHODS

KEY RESOURCES TABLE

REAGENT or RESOURCE	SOURCE	IDENTIFIER
**Biological samples**		
Ancient Skeletal element	this paper	Xaghra1
Ancient Skeletal element	this paper	Xaghra2
Ancient Skeletal element	this paper	Xaghra3
Ancient Skeletal element	this paper	Xaghra4
Ancient Skeletal element	this paper	Xaghra5
Ancient Skeletal element	this paper	Xaghra6
Ancient Skeletal element	this paper	Xaghra7
Ancient Skeletal element	this paper	Xaghra8
Ancient Skeletal element	this paper	Xaghra9
**Chemicals, peptides, and recombinant proteins**		
DNA extraction	Gamba et al.[12]	N/A
Library preparation	Gamba et al.[12]	N/A
AccuPrime Pfx	Invitrogen	Cat# 12344024
USER Enzyme	NEB	Cat# M5505L
**Critical commercial assays**		
Qubit dsDNA HS Assay Kit	Invitrogen	Q32854
D1000 ScreenTape	Agilent	Cat# 5067-5582
D1000 Reagents	Agilent	Cat# 5067-5583
**Deposited data**		
Human reference genome NCBI build 37, GRCh37	Genome Reference Consortium	https://www.ncbi.nlm.nih.gov/grc/human
Compiled modern and ancient comparison dataset 1240K, Human Origins and SGDP	N/A	https://reichdata.hms.harvard.edu/pub/datasets/
1000 Genomes Project Phase 3	The 1000 Genomes Project Consortium[78]	https://www.internationalgenome.org/category/phase-3/
Body mass index(BMI) meta-analysis data	UK Biobank	http://www.nealelab.is/uk-biobank
Maltese ancient DNA	This paper	http://www.ncbi.nlm.nih.gov/bioproject/778930<br>SRA: PRJNA778930
**Software and algorithms**		
cutadapt	Martin[79]	https://cutadapt.readthedocs.io/en/stable/#
AdapterRemoval	Schubert et al.[80]	https://adapterremoval.readthedocs.io/en/stable/
Burrow-Wheeler Aligner (BWA 0.7.5a)	Li and Durbin[81]	https://sourceforge.net/projects/bio-bwa/files/
SAMtools 1.7	Li et al.[82]	http://samtools.sourceforge.net/
Picard 1.101	N/A	https://broadinstitute.github.io/picard/
Qualimap 2.1.1	Okonechnikov et al.[83]	http://qualimap.conesalab.org/
Sex determination algorithm #1	Skoglund et al.[84]	https://github.com/pontussk/ry_compute
Sex determination algorithm #2	Cassidy et al.[6]	N/A
GATK	McKenna et al.[85]	https://gatk.broadinstitute.org/hc/en-us
PLINK 1.9	Chang et al.[86]	https://www.cog-genomics.org/plink/1.9
EIGENSOFT	Patterson et al.[87]	https://github.com/DReichLab/EIG
ADMIXTOOLS 7.0.2	Patterson et al.[88]	https://github.com/DReichLab/AdmixTools

(*Continued on next page*)

Continued

REAGENT or RESOURCE	SOURCE	IDENTIFIER
Beagle v.4.1	Browning and Browning[24,25]	https://faculty.washington.edu/browning/beagle/b4_1.html
SNPSift	Cingolani et al.[89]	https://pcingola.github.io/SnpEff/
bcftools 1.3	Li et al.[82]	https://sourceforge.net/projects/samtools/files/samtools/
SHAPEIT 2.r837	Delaneau et al.[90]	https://mathgen.stats.ox.ac.uk/genetics_software/shapeit/shapeit.html
ped-sim	Caballero et al.[91] and Campbell et al.[92]	https://github.com/williamslab/ped-sim
KING v.2.2.6	Manichaikul et al.[93]	https://kingrelatedness.com/
IBDseq vr1206	Browning and Browning[94]	https://faculty.washington.edu/browning/ibdseq.html
IBDNe 23Apr20.ae9	Browning and Browning[55]	https://faculty.washington.edu/browning/ibdne.html
hapROH 0.3a4	Ringbauer et al.[53]	https://pypi.org/project/hapROH/
EEMS	Petkova et al.[66]	https://github.com/dipetkov/eems
Chromopainter/fineSTRUCTURE v.2	Lawson et al.[65]	https://people.maths.bris.ac.uk/~madjl/finestructure/
Impute2	Howie et al.[20,21]	https://mathgen.stats.ox.ac.uk/impute/impute_v2.html

RESOURCE AVAILABILITY

Lead contact

Further information and requests for resources and reagents should be directed to and will be fulfilled by the lead contact: Daniel G. Bradley (dbradley@tcd.ie).

Materials availability

This study did not generate new unique reagents.

Data and code availability

- The FASTQ data have been deposited at (http://www.ncbi.nlm.nih.gov/bioproject/778930) and are publicly available as of the date of publication.
- This paper does not report original code.
- Any additional information required to reanalyze the data reported in this paper is available from the lead contact upon request.

EXPERIMENTAL MODEL AND SUBJECT DETAILS

Xagħra (Brochtorff) circle

The three sequenced samples all derive from the megalithic burial hypogeum on the Xagħra plateau between the temples of Ġgantija and Santa Verna, excavated between 1993 and 1994. The oldest sample (Xaghra6) derives from a deeper stratified area of stacked burials that also contained rich ceremonial objects higher in the stratigraphy. The two later samples were found in shallower deposits to the west. Xaghra5 was part of a display area of initially articulated human remains placed with portable figurines that was intentionally dismembered, most probably, at least in part to the slightly deeper location of Xaghra9 slightly to the north. Xaghra6 was placed as the use of the site began to intensify whereas the other two samples date to the period of most intensive activity some four hundred years later (c. 2500 BC).

METHOD DETAILS

Sampling and DNA extraction

For this project, 5 petrous bones and 4 teeth from the Xagħra Circle archaeological site in Malta have been processed in the clean room facilities of the Smurfit Institute, Trinity College, Dublin (Table 1). Full body suits, face masks, hairnets and gloves were worn during the work. All tools and surfaces were cleaned with bleach, DNA-ExitusPlus, ethanol and exposure to UV light. Samples were photographed extensively prior to any alterations, and were then exposed to UV light for 30 minutes on either side to remove surface contaminants. Sample drilling was carried out in a fume hood lined with bleached tinfoil. The surface of each bone was

cleaned using a drill bit. A triangular wedge section of the otic capsule region of each petrous bone and the root of each tooth were cut using a Dremel diamond wheel saw. Each sampled bone part was pulverised in a Mixer Mill MM 400 (Retsch). An aliquot of ∼0.1g of this bone powder was used for DNA extraction, and the rest of the powder was stored in a separate tube. The DNA extraction procedure followed the same protocol described in Yang et al.[95] with modifications presented elsewhere.[96] One sample subsequently sequenced at high coverage was re-extracted using an initial washing step by 0.5% bleach solution as described in Boessenkool et al..[97]

Radiocarbon analysis

Date ranges have been estimated using the 95% confidence interval of Bayesian chronological models of 117 radiocarbon dates from the site and their stratigraphic relationships.[7] The three sequenced samples all derive from the megalithic burial Circle on the Xagħra plateau between the temples of Ġgantija and Santa Verna, excavated between 1993 and 1994. The oldest sample (Xaghra6) derives from a deeper stratified area of stacked burials that also contained rich ceremonial objects higher in the stratigraphy. A burial in the same layer as Xaghra6 was radiocarbon dated to 2900–2650 BC (OxA-27837, 4198±26 BP).[7] The two later samples were found in shallower deposits to the west. Xaghra5 was part of a display area of initially articulated human remains placed with portable figurines that was intentionally dismembered, most probably, at least in part to the slightly deeper location of Xaghra9 slightly to the north. Xaghra6 was placed as the use of the site began to intensify whereas the other two samples date to the period of most intensive activity some four to five hundred years later, with 23 radiocarbon measurements from material associated with these samples spanning approximately 2550 to 2350 BC.

Library preparation

The initial screening of each sample and blank controls was performed by constructing a double-stranded DNA NGS library, priorly treated with Uracil-DNA-glycosylase (UDG), using the method outlined in Meyer and Kircher[98] and modified as described in Gamba et al. [12] Libraries were amplified with AccuPrime Pfx Supermix (Life Technology) using 12-14 cycles of PCR, assigned with unique indexes and quantified with a TapeStation 2200 (Agilent Technologies). The same libraries were also used for further amplifications required for high coverage sequencing.

DNA sequencing

The initial screening to assess the endogenous DNA was performed by sequencing all the libraries with the Illumina HiSeq 2500 platform (100bp SE) at Macrogen (Republic of Korea). Subsequently, 3 samples with high endogenous DNA were further sequenced to high coverage using the HiSeq 2500 Illumina platform (100bp SE) at Macrogen (Republic of Korea). One sample was further sequenced using NovaSeq (50bp PE) Illumina platforms at TrinSeq (Ireland).

QUANTIFICATION AND STATISTICAL ANALYSIS

Reads processing

For samples sequenced in single-end mode, reads were trimmed of their adapters and filtered based on their length using the software cutadapt v.1.9.1[79] (cutadapt -a AGATCGGAAGAGCACACGTCTGAACTCCAGTCAC -O 1 -m 34). For paired-end libraries, adapters were trimmed and reads were filtered using AdapterRemoval v2.1.1[80] (–trimns –trimqualities –minquality 25 –collapse). Reads that passed these qualities and length filters were aligned to the human reference genome (hg19/GRCh37) with the mitochondrial sequence replaced by the Revised Cambridge Reference Sequence (rCRS, NC_012920.1) using the software BWA v.0.7.5a[81] with relaxed parameters (-l 1024 -n 0.01 -o 2). Aligned reads that came from PCR duplication or with a mapping quality below 20 were removed using the software SAMtools v.1.7[82] and Picard Tools v.1.101 (http://broadinstitute.github.io/picard/). The coverage of each completed aligned file was calculated using the tool Qualimap v.2.1.1.[83] Indels were locally realigned using The RealignerTargetCreator and IndelRealigner tools from GATK v.2.4.[85] Additionally 2bp were soft clipped at the start and end of each read.

Contamination estimation and sex determination

To determine the sex of each sample we applied two methods, one outlined in[84] and the other described in Cassidy et al.[6] In both methods, the amount of reads aligned on the X chromosome versus the autosomal genome was used to estimate the sex of an individual together with a confidence interval. We only considered sex assignments where both methods agreed. For three Maltese samples analysed in this study we estimated contamination using the haploid information contained in the mitochondrial genome and in the X chromosome for two males, applying the same method outlined in.[15]

Population structure analysis

Pseudohaploid genotypes were called at approximately 600,000 autosomal sites from the Human Origins panel[27] for the same set of ancient samples used in the IBD analyses plus 19 other ancient samples representative of hunter-gatherers, Bronze Age and Neolithic farmers populations.[13,14,18,99] Read bases were determined at each site using the Pileup tool from GATK v2.4,[85] filtered for a quality of 30, with bases not matching either the reference or alternate allele removed. A single base was then randomly selected to generate the pseudohaploid genotype. This ancient dataset was then merged with a subset of the Human Origins panel from

Western Eurasia using the software PLINK v1.9.[86] A Principal Component Analysis (PCA) was then carried out on the 604 modern individuals from Human Origins, with the genetic variation of the ancient samples projected onto this using the SmartPCA v.16000 algorithm implemented in EIGENSOFT[87,100] with parameters (killr2: YES, r2thresh: 0.2, numoutlieriter: 0, lsqproject: YES, autoshrink: YES) (Figure S1).

F-statistics

Using the same set of ancient samples described in the previous paragraph and transversion sites only from the "1240K" panel,[13,14,23] we estimated the amount of drift that the Maltese shared with each other population using the *outgroup-f3* statistics[88,101] method implemented in the ADMIXTOOLS package v.7.0.2.[88] This analysis was carried out in the form of *f3*(Mbuti; Ancient Maltese, X) where X represents different populations tested (Data S2F; Figure S2). The outgroup population, Mbuti, is represented by four individuals collected from the SGDP dataset.[102]

To test for Admixture with African populations we used *D* statistics.[103] Four ancient North African representatives were selected from Fregel et al.[104] (Data S2A). Tests were constructed in the form of: *D*(Chimp, Ancient North-Africa, Malta Late Neolithic, X) where X represents Neolithic populations that fall close in the PCA to the ancient Maltese (Figure S1; Data S2D).

Similarly to test for admixture between the Maltese and Caucasus hunter-gatherer (CHG) or Steppe populations we built our *D* statistics test in the form of *D*(Mbuti, CHG/Yamnaya, Malta Late Neolithic, X). In this analysis the CHG population is represented by two individuals published in Jones et al.[18] (Data S2B and S2C).

To estimate the amount of WHG ancestry we used the method qpAdm Haak et al.[14] We first divided the individuals into groups according to their archaeological site of origin. Each group was furthermore subdivided in bins of 1000 years and only sub-groups with at least 2 individuals were considered for this analysis. The reference group was comprised of the following genomes: (Mbuti.DG, Ust-Ishim, MA1, Villabruna, GoyetQ116-1, Han.DG, Papuan.DG, Mixe.DG, Karitiana.DG, AHG, Iran_Neolithic, CHG, EHG). The source population are Anatolian_Neolithic represented by individuals from Barcin and WHG individuals represented by Loschbour and KO1 (Data S2E). Only groups with a p-value higher than 0.05 were included in Figure S3.

Genotype imputation

From samples that had been screened using an in-solution target capture method we selected 231 published genomes for imputation with a reported coverage on target regions of at least 0.6X and 650K SNPs called from the 1240K panel. To increase the number of samples from Neolithic Sardinia we also included 4 samples with a coverage higher than 0.6X and at least 460K SNPs safely called from the 1240K panel. Before imputation we selected a set of approximately 6.2 million SNPs to be called on our target dataset using the 1000 Genomes Project (1000G) resource as reference, filtered for individuals of African origin (defined with the AFR label) and with a minor allele frequency of 5%. Variants were called using the tool UnifiedGenotyper in GATK v2.4[85] program with parameters (–output_mode EMIT_ALL_SITES, –genotyping_mode GENOTYPE_GIVEN_ALLELES). The VCF files created were then split first by chromosome and then by windows of 1 Mb. Genotype imputation was performed on approximately 28 million variants using the tool Beagle v.4.1[24,25] with a reference dataset of 1843 modern individuals of non-African origin from the 1000 Genomes project. The program was run in multi-thread mode taking advantage of the Irish Centre for High-End Computing (ICHEC) cluster. The genetic map used was taken from the Beagle website (http://bochet.gcc.biostat.washington.edu/beagle/genetic_maps/). The imputed VCF files were filtered for SNPs only and genotype probability of 0.95 using bcftools v.1.3[82] and PLINK v1.9 (–vcf-min-gp 0.95)[86] obtaining 25.8 million variants.

After completion of imputation, four samples with high genotype missingness (>=0.1) were removed from subsequent analyses. Separately we also selected 120 WGS samples with a coverage of at least 0.4X to impute using the Software Impute2.[20,21] For these samples, and similarly to the SNP capture imputation, variants were called from the 1000 Genomes project[78] using the tool UnifiedGenotyper in GATK v.2.4[85] using the same parameters. The whole 1000 Genomes project dataset was used as reference for the genotype imputation. Prior to imputation transition SNPs were excluded from this dataset resulting in calling of approximately 28 million. The VCF file was then split first by chromosome and then in windows containing 15000 markers. For each input file, the program Impute2 was called using the parameters (-Ne 20000 -buffer 500 -allow_large_regions -k 400 -k_hap 2000). After imputation we filtered for genotype probability higher than 0.99 (GP > 0.99) resulting in 77.8 million SNPs.

Finally this combined resource was merged with 21 low coverage shotgun sequenced genomes which had been previously imputed.[22] Between WGS and SNP capture samples we obtained a final resource of 357 unique imputed diploid genomes (Data S1A; Figure S6).

After merging these resources we then tested for differences in genotype missingness between datasets. To do so we first considered a set of 12 million SNPs common across all three datasets. We then calculated the missingness for each dataset and averaged across samples. We observed a genotype missing for the SNP capture and WGS imputed respectively of 12% and 13.5%.

ROH and inbreeding analysis

To estimate the inbreeding coefficients of our imputed samples, we used a measure based on the proportion of the genome that is homozygous-by-descent (runs of homozygosity that are identical by descent), as employed in Cassidy et al.,[6] and labelled here as F_{ROH}. Separately, the hunter-gatherer and Neolithic farmer datasets were filtered for genotypes missingness and minor allele frequency using PLINK v1.9 (–geno 0.02, –maf 0.05, –indep 50 2 2) obtaining respectively 51,289 SNPs and 41,426 SNPs. Using this set of SNPs we then identified ROH segments using PLINK v1.9. with the same parameters used in Gazal et al.[105]

CellPress
OPEN ACCESS

(–homozyg-window-het 0 –homozyg-snp 50 –homozyg-kb 1 –homozyg-density 5000 –homozyg-gap 5000). Physical measures were converted to centiMorgans (cM). The total length of the genome in ROH above this threshold divided by the length of the autosomal part was used to estimate the F_{ROH} coefficients.[106] To assess the concordance between samples imputed from different sources we compared F_{ROH} estimates obtained from imputed SNP capture with those calculated using imputed data from WGS data available for the same samples. We considered the same set of SNPs in both data types. For two samples that were whole genome screened and where the coverage was sufficiently high we also estimated the F_{ROH} coefficients using diploid genotype calls. For these two samples we applied the same protocol described in the imputation accuracy paragraph. In brief diploid genotypes with a depth below 10 or higher than 30 and a quality below 30 were excluded. As shown in Figure S4 there is no visible deviation of substance between the measures.

For the sample, Xaghra9, which has sufficient coverage, we ran the software ROHan[52] to validate our inbreeding results. As suggested by the software we first used the program bam2prof with different threshold values (–length 5, 10, 15, 20) to account for post-mortem deamination damage. We then run the program rohan using transversion only (–tvsonly).

Pedigree simulation

To better understand the degree of relatedness between the parents of inbred samples we simulated different pedigree scenarios using dummy genotypes. We started from the same dataset described in the previous paragraph and we filtered for genotype missingness and minor allele frequency. This filtered resource was split by chromosome and then re-phased using SHAPEIT v.2.r837.[90] After phasing we filtered for linkage disequilibrium with plink using (–indep 50 2 2) and selected a common set of SNPs. 21 Irish imputed individuals published in Cassidy et al.[6] were selected from this dataset as founders to build the simulated pedigrees. This set of founders were not influenced by inbreeding, relatedness, population structure, or recent change in population size. This dataset was then used as input for the software ped-sim[91,92] with a refined genetic map taken from Bhérer et al. [107] Three different inbreeding scenarios were tested:

- First degree: siblings and parent-offspring
- Second degree: uncle-niece/aunt-nephew and grandparent-grandchild
- Third degree: first cousins and great aunt-great nephew/great uncle-great niece

Each of these scenarios was simulated 400 times using random sampled founders. ROH segments were found using PLINK with the same parameters described in the previous section (–homozyg-window-het 0 –homozyg-snp 50 –homozyg-kb 1 –homozyg-density 5000 –homozyg-gap 5000) and inbreeding coefficients estimates were also obtained using the same pipeline for both simulated and real genomes.

IBD analysis

In this work, we applied the software IBDSeq vr1206[94] to the unphased dataset to identify segments of the genome inherited by recent common ancestors (identical by descent) in European Neolithic samples. Genotype missingness and minor allele frequency filters were applied to the imputed dataset using the software PLINK v.1.9 (–geno 0.02, –maf 0.05). Related individuals with a relatedness estimated by the software KING v.2.2.6[105] higher than 4th degree relatives were also removed from analyses obtaining 258 unrelated samples. Filtered files in PLINK format were converted to VCF using the option (–vcf) in PLINK v1.9. and used as input to the program IBDSeq with parameters (errormax=0.005 and LOD >= 3;[108]). IBD segments shorter than 2 cM were excluded following the advice of Browning and Browning.[94]

To test that no systematic bias was present between types of data, we compared the results obtained from those samples where it was possible to impute genome wide calls using both WGS and SNP capture data. We used a common set of SNPs for both data types that were pruned for genotype missingness and minor allele frequency, obtaining approximately 900 thousands markers per comparison. This set of common SNPs was then used to calculate the total amount of IBD that each sample type, WGS or SNP capture, shared with the rest of the dataset. As shown in Figure S4 correlation and variation around the 1:1 plot line indicate no systematic bias between captured and WGS imputed data

Population size estimates

To estimate the effective population size we used the IBD information obtained from IBDSeq as an input for the software IBDNe v.23Apr20.ae9.[55] This software was run for 50 generations with default settings and only for groups that shared at least 90 IBD segments longer than 2cM. An estimate of population size for each group was calculated by taking the harmonic mean over 25 generations (from 5 to 30).

Separately we also estimated the effective population size of our Maltese group using the software hapROH v0.3a4.[53] First we excluded the highly inbred sample Xaghra9 from this group. For the remaining two imputed samples(Xaghra5 and Xaghra6), diploid genotypes were downsampled to ‘‘1240K’’ SNPs panel and ROH were called with plink similar to what is described above (–homozyg-window-het 0 –homozyg-snp 50 –homozyg-kb 1 –homozyg-density 5000 –homozyg-gap 5000). For each of the two Maltese samples the ROH results were then used to estimate the effective population size using the function ‘‘MLE_ROH_Ne’’ from the hapROH package using the parameters (min_len=4, max_len=20, ne=10000, bin_range=[0.04, 0.5], nbins=1000, error_model=False).

Chromopainter/fineSTRUCTURE

To investigate fine-scale population structure in our imputed dataset we used the software fineSTRUCTURE v2.[65] The same set of unrelated samples used in the IBDseq analysis were used for this analysis. These ancient imputed samples were filtered for genotype missingness and minor allele frequency using the software PLINK v.1.9. with parameters (–geno 0 –maf 0.01). After filtering, approximately 220K SNPs were used to phase the genotypes using the software SHAPEIT v.2.r778.[109] For each chromosome separately we ran Chromopainter first to estimate the "Ne" and "mu" parameters using 10 expectation maximization iteration (-i 10). These parameters were then used to paint each individual against all the others (-a 0 0). Finally we used "Chromocombine" to merge the painting information from each chromosome and obtain the normalization parameter "c".

The estimated matrix of chunk counts (Data S1C) obtained from Chromocombine was then used as input to the fineSTRUCTURE algorithm. This program was run using 1,000,000 burnin and sampling iterations with sampling every 1000 iterations for the MCMC. Following the method described in Leslie et al.[75] we then extracted the state with the highest posterior probability and performed an additional 100,000 burn-in iterations using the maximum concordance method to obtain the final tree. The information about the optimal number of groups and the cluster assignment of each sample was taken from the file ".tree" generated by the program.

Estimated effective migration surface

To visualize how geographical barriers affected migration between populations we used the software EEMS.[66] The same set of non-related ancient samples used for the IBD analyses were used to generate a pairwise dissimilarity matrix using the bed2diffs v.2. program. EEMS was initially run using 500 demes with MCMC chains parameters of 100,000 burn-in and 200,000 sampling iterations. This run was repeated 10 times using different random seeds. The run with the highest likelihood was then selected for further refinement using the same number of demes and MCMC settings of 1000,000 burn-in and 2000,000 sampling iterations.

BMI analysis

To investigate the distribution of body mass index across European Neolithic populations we calculated the polygenic risk score (PRS) for 247 individuals using the summary statistics calculated by the Neale Lab (http://www.nealelab.is/uk-biobank) using the UK BioBank resource. Prior to obtaining the PRS information we filtered individuals with more than 30% of BMI SNPs missing. We did not allow missing genotypes to be present in this analysis. SNPs in this dataset were filtered using a clumping/threshold approach through the software PLINK 1.9 with parameters (–clump-p1 0.01 –clump-kb 1000 –clump-r2 0.1). After filtering we obtained approximately 12 thousands SNPs that we used to compute the PRS in 247 ancient samples using the –score option in plink (Figure S7).

Current Biology, Volume *32*

Supplemental Information

Ancient Maltese genomes and the genetic geography of Neolithic Europe

Bruno Ariano, Valeria Mattiangeli, Emily M. Breslin, Eóin W. Parkinson, T. Rowan McLaughlin, Jess E. Thompson, Ronika K. Power, Jay T. Stock, Bernardette Mercieca-Spiteri, Simon Stoddart, Caroline Malone, Shyam Gopalakrishnan, Lara M. Cassidy, and Daniel G. Bradley

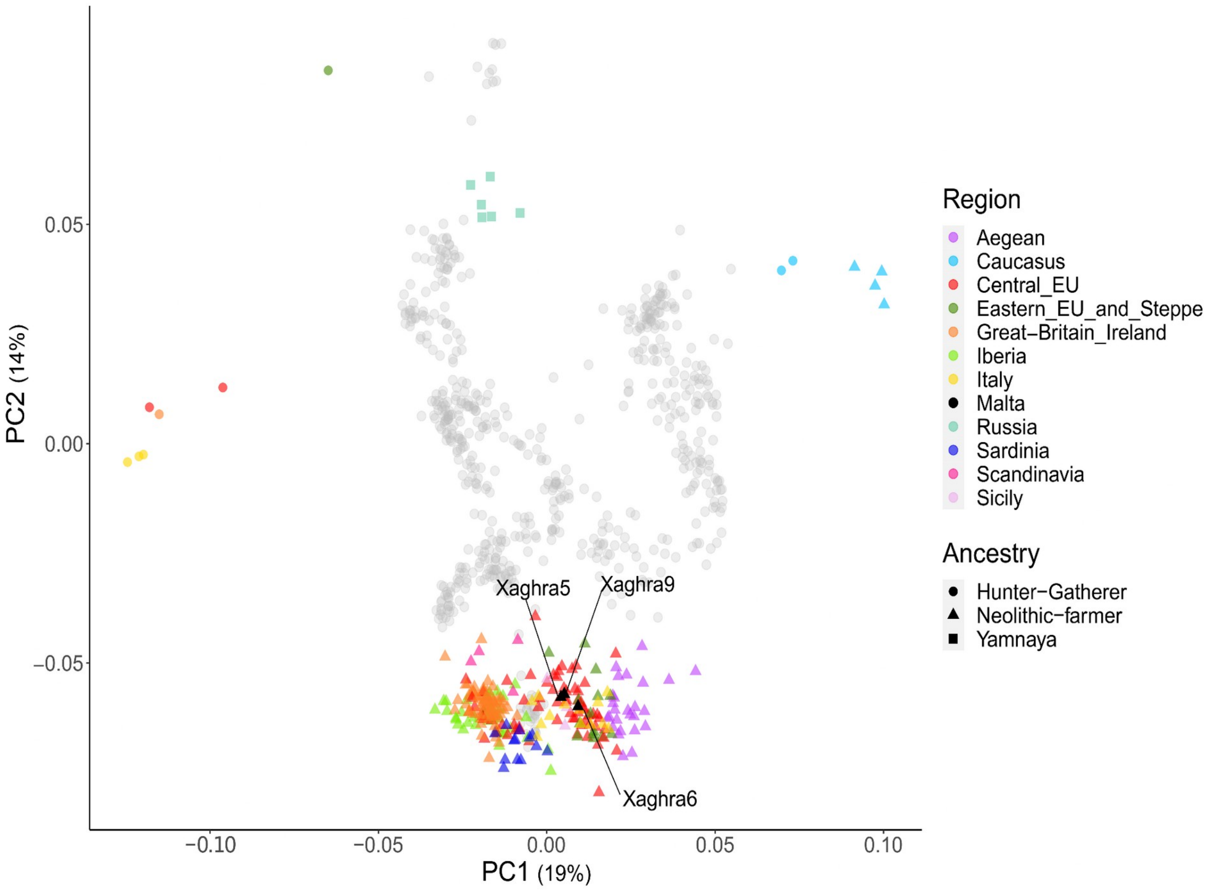

Figure S1: PCA for ancient Maltese and other ancient European populations. **Related to Star Methods and Data S2.** Maltese genomes as well as other published Neolithic and Mesolithic genomes were projected onto a principal component analysis plot using the Human Origins dataset as a modern reference. For each principal component the percentage of variance explained is indicated in parentheses. The ancient Maltese samples group together with Central and Southern European Neolithic genomes.

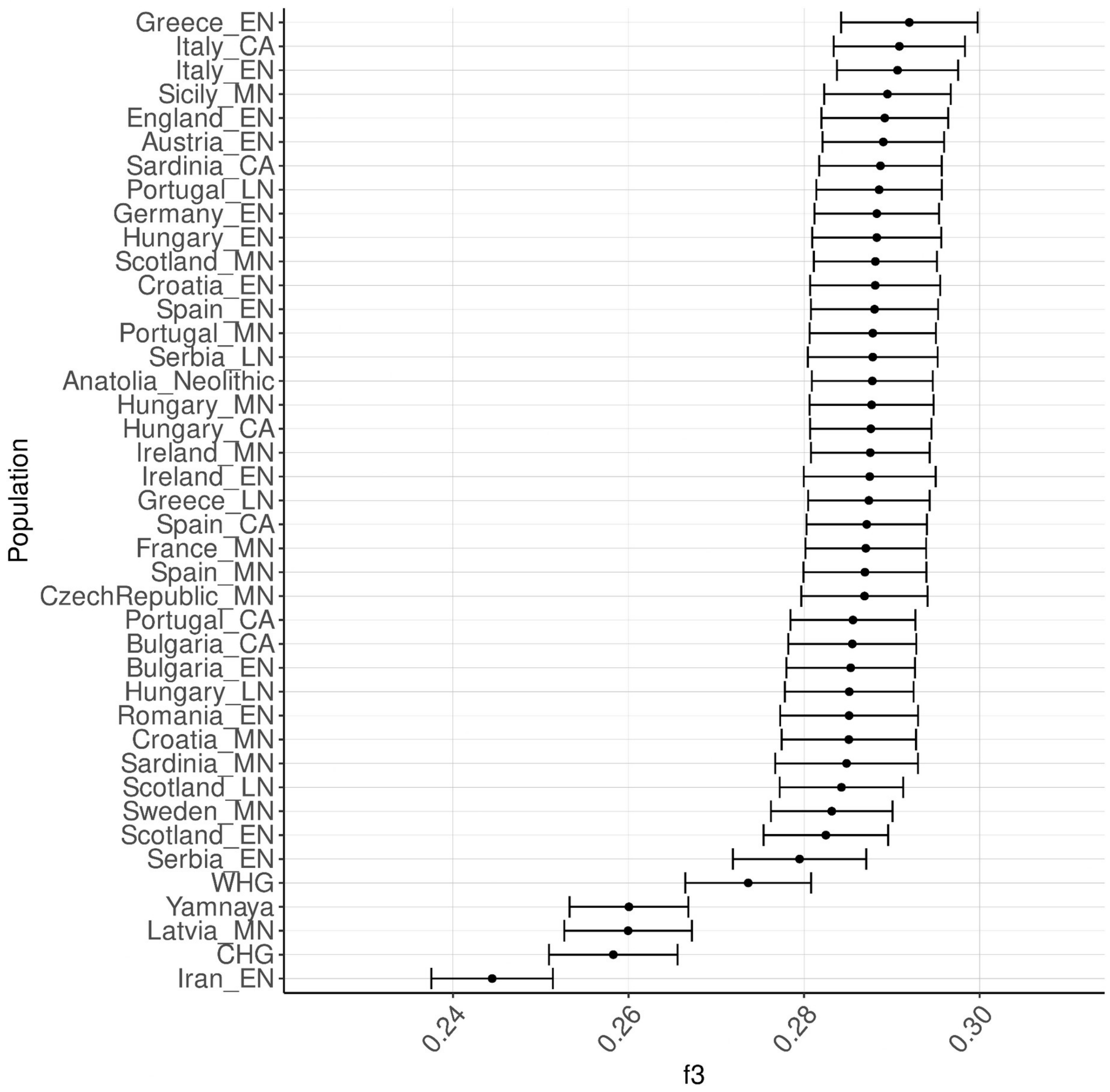

Figure S2: Shared drift measured using the outgroup-f3 statistics. **Related to Star Methods and Data S2F.** The results using outgroup-f3 statistics represented by circles with error bars in the form (Mbuti; X, Malta Neolithic) show the Maltese being closer to early Neolithic individuals from Greece and the Italian peninsula. (EN=Early Neolithic, MN=Middle Neolithic, LN=Late Neolithic, CA=Copper Age, WHG=Western hunter-gatherer, CHG=Caucasus hunter-gatherer, EHG=Eastern hunter-gatherer). The Anatolian Neolithic population is represented by individuals from Marmara, Barcın.

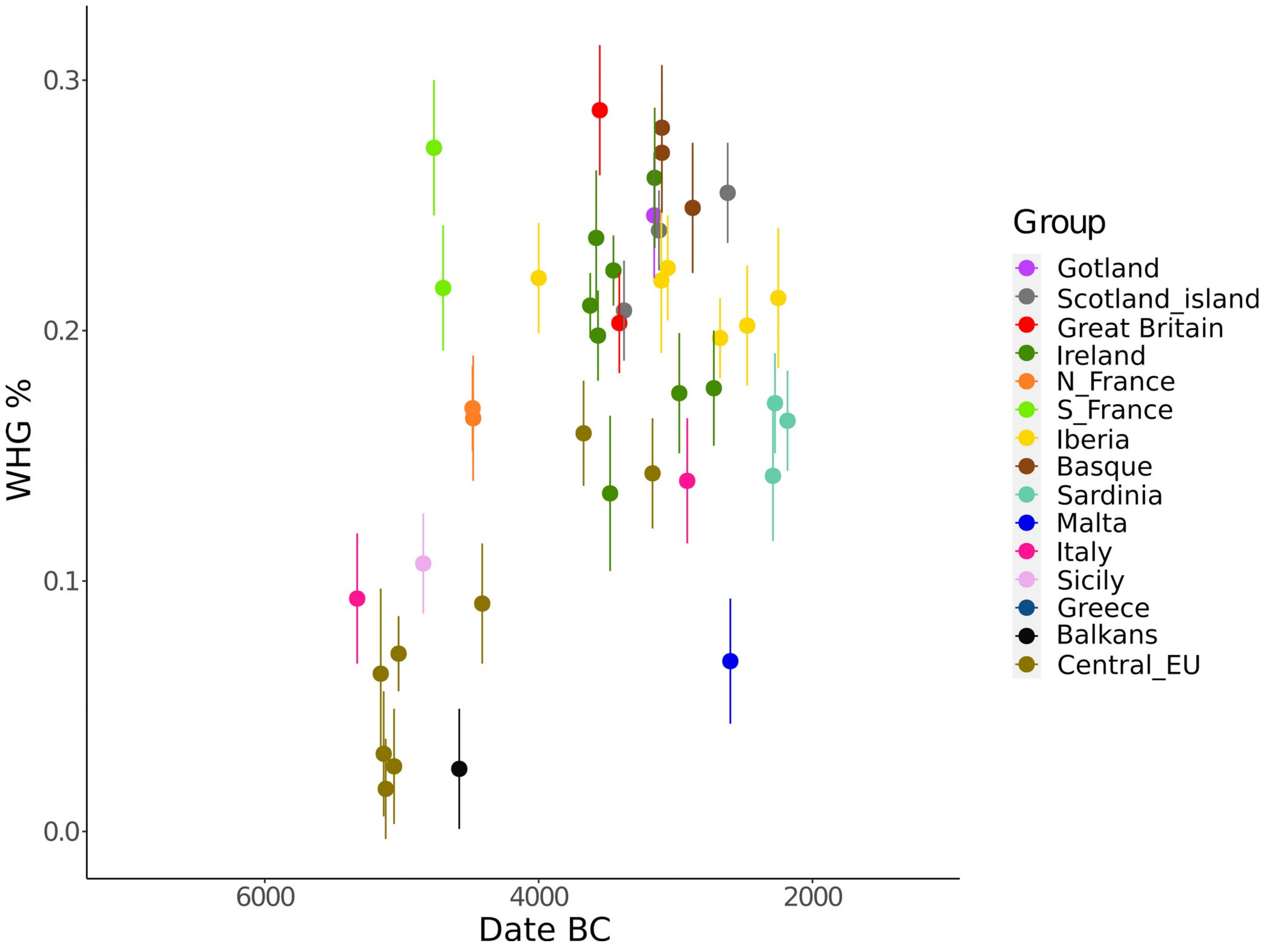

Figure S3: Temporal distribution of hunter-gatherer ancestry in Neolithic Europeans. Related to Star Methods and Data S2E. qpAdm was used to measure the quantity of Western Hunter-gatherer (WHG) populations, represented by the individuals KO1 and Loschbour, present in European Neolithic populations. Each point represents with its error bar a group with at least 2 individuals from an archaeological site and time period. The WHG percentages are reported with 95% error bars.

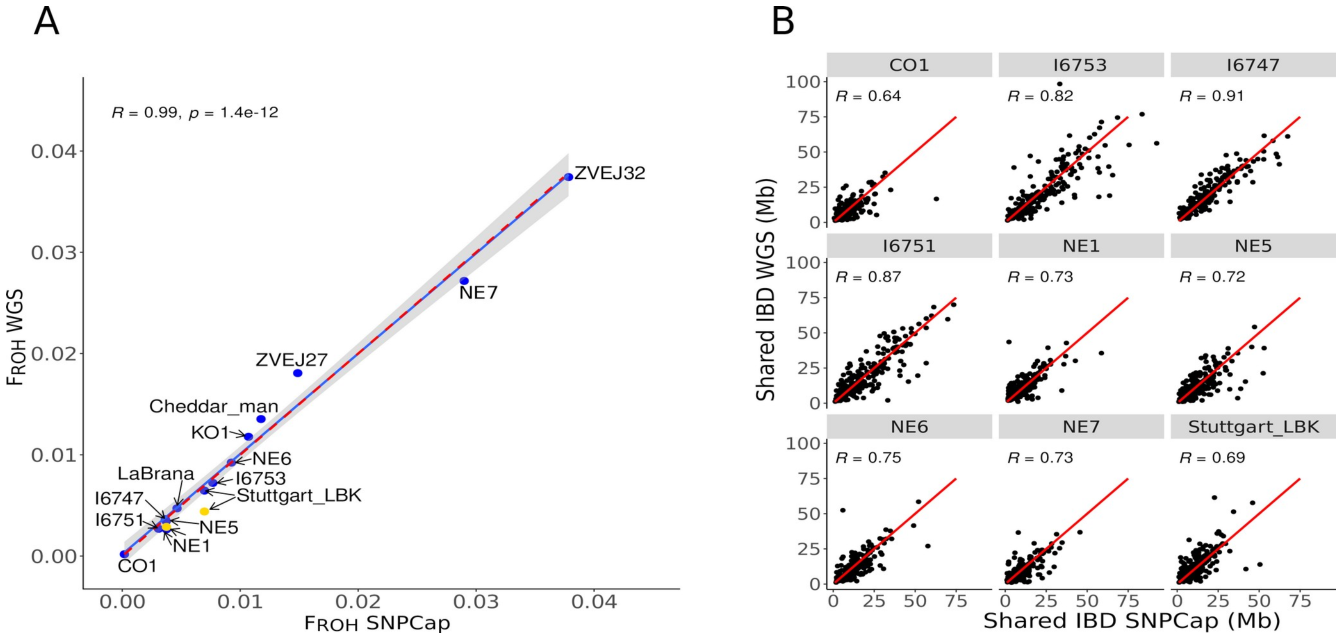

Figure S4: Comparisons of IBD and F_{ROH} estimates from imputed WGS and SNP captured data. Related to Star Methods and Data S1B, S1D. (A) F_{ROH} were compared between imputations of WGS and SNP captured data (respectively "F_{ROH} WGS" and "F_{ROH} SNPCap") where these are available from the same samples. These correlate with a P-value lower than and the regression line (coloured blue) error margins overlap with the 1:1 plot (red line). Also two yellow coloured points denote where three genomes (NE1, Stuttgart_LBK) also had WGS F_{ROH} estimates available from high coverage SNP calls - these are plotted Vs F_{ROH} SNPCap. (B) Plots of IBD sharing values involving each of nine Neolithic samples for which WGS and SNP capture - based estimates are available. For each, total estimates of the genome shared with all other Neolithic samples is plotted alternately using the two different data sets. The WGS and SNP-derived values correlate significantly (each at $p < 10^{-15}$) and vary around the 1:1 plotline, drawn in red.

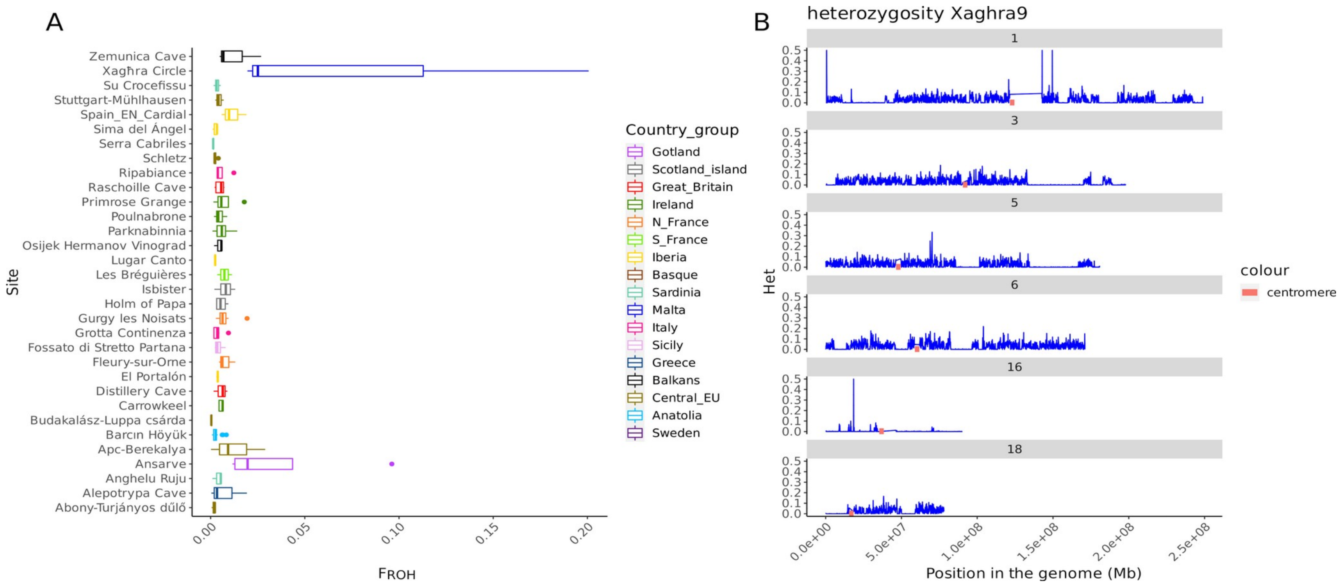

Figure S5: Inbreeding F_{ROH} coefficient estimates and chromosome heterozygosity plots for Xaghra9. Related to Figure 2A and Data S1D. (A) F_{ROH} coefficients within sites with a minimum of 3 individuals. The Maltese possess the highest median inbreeding coefficient followed by values from Gotland. (B) The average heterozygosity is plotted for Xaghra9 using a window size of 100 kb and illustrates examples of long genome tracts of homozygosity in selected chromosomes.

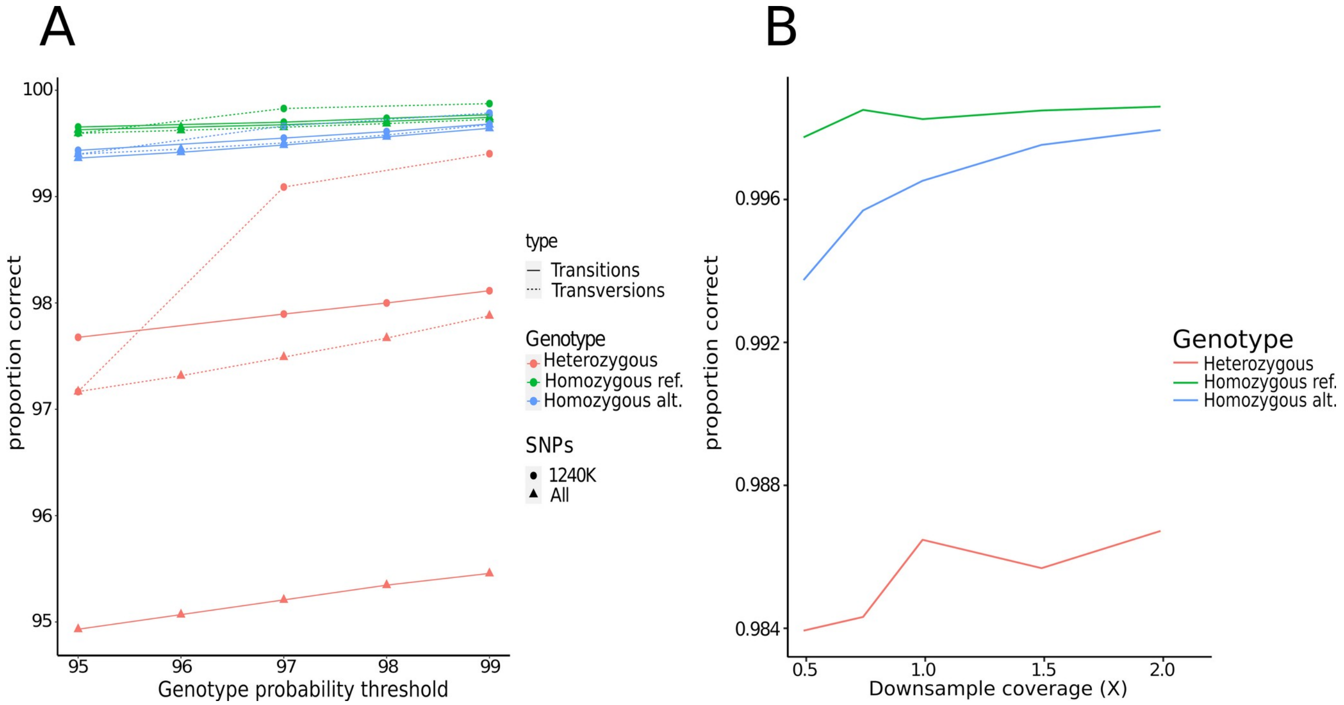

Figure S6: Accuracy of imputed genotypes. **Related to Star Methods.** (A) Genotypes imputed from a SNP captured genome (LBK) compared against those called from its high coverage genome sequence using the software SNPSift[S43]. Heterozygous and transversion SNPs show overall lower accuracy compared to homozygous and transition imputed SNPs. The SNPs corresponding to the 1240K positions were predicted overall with higher accuracy compared to other variants. (B) Genotypes imputed from a downsampled WGS genome (LBK) also here compared against its high coverage genome sequence. Only transversions are considered in this analysis and heterozygous calls show overall less accuracy than homozygous ones. Overall the percentage of safely imputed genotypes remains high for each genotype class across different probability thresholds.

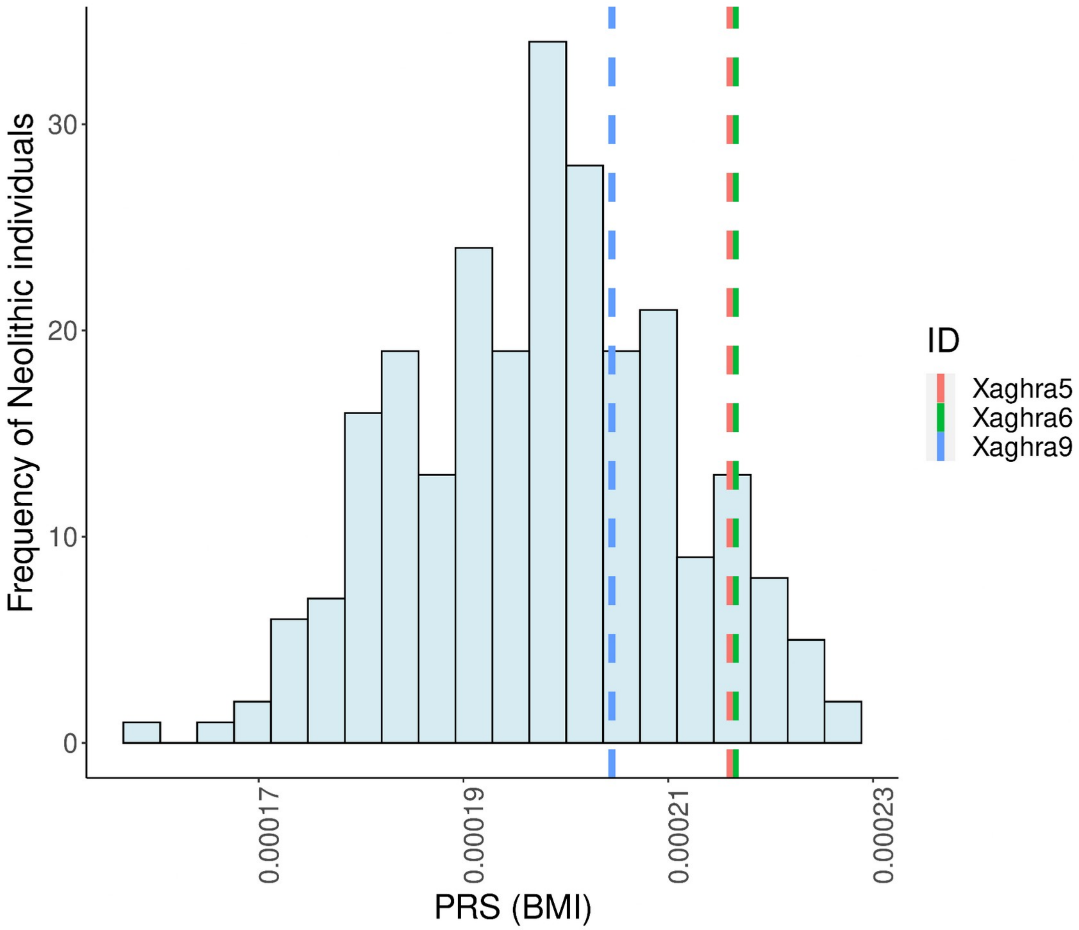

Figure S7: Body mass index in ancient Maltese. **Related to Star Methods.** Polygenic risk score (PRS) information calculated from the UK Biobank dataset was used to estimate body mass index (BMI) of the ancient Maltese together with 247 European Neolithic samples. None of the former showed extreme values.

Supplemental References

S1. Keller, A., Graefen, A., Ball, M., Matzas, M., Boisguerin, V., Maixner, F., Leidinger, P., Backes, C., Khairat, R., Forster, M., et al. (2012). New insights into the Tyrolean Iceman's origin and phenotype as inferred by whole-genome sequencing. Nat. Commun. *3*, 698.

S2. Lazaridis, I., Patterson, N., Mittnik, A., Renaud, G., Mallick, S., Kirsanow, K., Sudmant, P.H., Schraiber, J.G., Castellano, S., Lipson, M., et al. (2014). Ancient human genomes suggest three ancestral populations for present-day Europeans. Nature *513*, 409–413.

S3. Skoglund, P., Malmström, H., Omrak, A., Raghavan, M., Valdiosera, C., Günther, T., Hall, P., Tambets, K., Parik, J., Sjögren, K.-G., et al. (2014). Genomic diversity and admixture differs for Stone-Age Scandinavian foragers and farmers. Science *344*, 747–750.

S4. Gamba, C., Jones, E.R., Teasdale, M.D., McLaughlin, R.L., Gonzalez-Fortes, G., Mattiangeli, V., Domboróczki, L., Kővári, I., Pap, I., Anders, A., et al. (2014). Genome flux and stasis in a five millennium transect of European prehistory. Nat. Commun. *5*, 5257.

S5. Seguin-Orlando, A., Korneliussen, T.S., Sikora, M., Malaspinas, A.-S., Manica, A., Moltke, I., Albrechtsen, A., Ko, A., Margaryan, A., Moiseyev, V., et al. (2014). Paleogenomics. Genomic structure in Europeans dating back at least 36,200 years. Science *346*, 1113–1118.

S6. Raghavan, M., Skoglund, P., Graf, K.E., Metspalu, M., Albrechtsen, A., Moltke, I., Rasmussen, S., Stafford, T.W., Jr, Orlando, L., Metspalu, E., et al. (2014). Upper Palaeolithic Siberian genome reveals dual ancestry of Native Americans. Nature *505*, 87–91.

S7. Fu, Q., Li, H., Moorjani, P., Jay, F., Slepchenko, S.M., Bondarev, A.A., Johnson, P.L.F., Aximu-Petri, A., Prüfer, K., de Filippo, C., et al. (2014). Genome sequence of a 45,000-year-old modern human from western Siberia. Nature *514*, 445–449.

S8. Olalde, I., Allentoft, M.E., Sánchez-Quinto, F., Santpere, G., Chiang, C.W.K., DeGiorgio, M., Prado-Martinez, J., Rodríguez, J.A., Rasmussen, S., Quilez, J., et al. (2014). Derived immune and ancestral pigmentation alleles in a 7,000-year-old Mesolithic European. Nature *507*, 225–228.

S9. Mathieson, I., Lazaridis, I., Rohland, N., Mallick, S., Patterson, N., Roodenberg, S.A., Harney, E., Stewardson, K., Fernandes, D., Novak, M., et al. (2015). Genome-wide patterns of selection in 230 ancient Eurasians.

Nature *528*, 499–503.

S10. Jones, E.R., Gonzalez-Fortes, G., Connell, S., Siska, V., Eriksson, A., Martiniano, R., McLaughlin, R.L., Gallego Llorente, M., Cassidy, L.M., Gamba, C., et al. (2015). Upper Palaeolithic genomes reveal deep roots of modern Eurasians. Nat. Commun. *6*, 8912.

S11. Olalde, I., Schroeder, H., Sandoval-Velasco, M., Vinner, L., Lobón, I., Ramirez, O., Civit, S., García Borja, P., Salazar-García, D.C., Talamo, S., et al. (2015). A Common Genetic Origin for Early Farmers from Mediterranean Cardial and Central European LBK Cultures. Mol. Biol. Evol. *32*, 3132–3142.

S12. Günther, T., Valdiosera, C., Malmström, H., Ureña, I., Rodriguez-Varela, R., Sverrisdóttir, Ó.O., Daskalaki, E.A., Skoglund, P., Naidoo, T., Svensson, E.M., et al. (2015). Ancient genomes link early farmers from Atapuerca in Spain to modern-day Basques. Proc. Natl. Acad. Sci. U. S. A. *112*, 11917–11922.

S13. Allentoft, M.E., Sikora, M., Sjögren, K.-G., Rasmussen, S., Rasmussen, M., Stenderup, J., Damgaard, P.B., Schroeder, H., Ahlström, T., Vinner, L., et al. (2015). Population genomics of Bronze Age Eurasia. Nature *522*, 167–172.

S14. Hofmanová, Z., Kreutzer, S., Hellenthal, G., Sell, C., Diekmann, Y., Díez-Del-Molino, D., van Dorp, L., López, S., Kousathanas, A., Link, V., et al. (2016). Early farmers from across Europe directly descended from Neolithic Aegeans. Proc. Natl. Acad. Sci. U. S. A. *113*, 6886–6891.

S15. Mallick, S., Li, H., Lipson, M., Mathieson, I., Gymrek, M., Racimo, F., Zhao, M., Chennagiri, N., Nordenfelt, S., Tandon, A., et al. (2016). The Simons Genome Diversity Project: 300 genomes from 142 diverse populations. Nature *538*, 201–206.

S16. Kılınç, G.M., Omrak, A., Özer, F., Günther, T., Büyükkarakaya, A.M., Bıçakçı, E., Baird, D., Dönertaş, H.M., Ghalichi, A., Yaka, R., et al. (2016). The Demographic Development of the First Farmers in Anatolia. Curr. Biol. *26*, 2659–2666.

S17. Fu, Q., Posth, C., Hajdinjak, M., Petr, M., Mallick, S., Fernandes, D., Furtwängler, A., Haak, W., Meyer, M., Mittnik, A., et al. (2016). The genetic history of Ice Age Europe. Nature *534*, 200–205.

S18. Cassidy, L.M., Martiniano, R., Murphy, E.M., Teasdale, M.D., Mallory, J., Hartwell, B., and Bradley, D.G. (2016). Neolithic and Bronze Age migration to Ireland and establishment of the insular Atlantic genome. Proc. Natl. Acad. Sci. U. S. A. *113*, 368–373.

S19. Broushaki, F., Thomas, M.G., Link, V., López, S., van Dorp, L., Kirsanow, K., Hofmanová, Z., Diekmann, Y., Cassidy, L.M., Díez-Del-Molino, D., et al.

(2016). Early Neolithic genomes from the eastern Fertile Crescent. Science *353*, 499–503.

S20. González-Fortes, G., Jones, E.R., Lightfoot, E., Bonsall, C., Lazar, C., Grandal-d'Anglade, A., Garralda, M.D., Drak, L., Siska, V., Simalcsik, A., et al. (2017). Paleogenomic Evidence for Multi-generational Mixing between Neolithic Farmers and Mesolithic Hunter-Gatherers in the Lower Danube Basin. Curr. Biol. *27*, 1801–1810.e10.

S21. Jones, E.R., Zarina, G., Moiseyev, V., Lightfoot, E., Nigst, P.R., Manica, A., Pinhasi, R., and Bradley, D.G. (2017). The Neolithic Transition in the Baltic Was Not Driven by Admixture with Early European Farmers. Curr. Biol. *27*, 576–582.

S22. Lipson, M., Szécsényi-Nagy, A., Mallick, S., Pósa, A., Stégmár, B., Keerl, V., Rohland, N., Stewardson, K., Ferry, M., Michel, M., et al. (2017). Parallel palaeogenomic transects reveal complex genetic history of early European farmers. Nature *551*, 368–372.

S23. Martiniano, R., Cassidy, L.M., Ó'Maoldúin, R., McLaughlin, R., Silva, N.M., Manco, L., Fidalgo, D., Pereira, T., Coelho, M.J., Serra, M., et al. (2017). The population genomics of archaeological transition in west Iberia: Investigation of ancient substructure using imputation and haplotype-based methods. PLoS Genet. *13*, e1006852.

S24. Sikora, M., Seguin-Orlando, A., Sousa, V.C., Albrechtsen, A., Korneliussen, T., Ko, A., Rasmussen, S., Dupanloup, I., Nigst, P.R., Bosch, M.D., et al. (2017). Ancient genomes show social and reproductive behavior of early Upper Paleolithic foragers. Science *358*, 659–662.

S25. Mathieson, I., Alpaslan-Roodenberg, S., Posth, C., Szécsényi-Nagy, A., Rohland, N., Mallick, S., Olalde, I., Broomandkhoshbacht, N., Candilio, F., Cheronet, O., et al. (2018). The genomic history of southeastern Europe. Nature *555*, 197–203.

S26. Fregel, R., Méndez, F.L., Bokbot, Y., Martín-Socas, D., Camalich-Massieu, M.D., Santana, J., Morales, J., Ávila-Arcos, M.C., Underhill, P.A., Shapiro, B., et al. (2018). Ancient genomes from North Africa evidence prehistoric migrations to the Maghreb from both the Levant and Europe. Proc. Natl. Acad. Sci. U. S. A. *115*, 6774–6779.

S27. Olalde, I., Brace, S., Allentoft, M.E., Armit, I., Kristiansen, K., Booth, T., Rohland, N., Mallick, S., Szécsényi-Nagy, A., Mittnik, A., et al. (2018). The Beaker phenomenon and the genomic transformation of northwest Europe. Nature *555*, 190–196.

S28. Günther, T., Malmström, H., Svensson, E.M., Omrak, A., Sánchez-Quinto, F., Kılınç, G.M., Krzewińska, M., Eriksson, G., Fraser, M., Edlund, H., et al. (2018). Population genomics of Mesolithic Scandinavia: Investigating early

postglacial migration routes and high-latitude adaptation. PLoS Biol. *16*, e2003703.

S29. Harney, É., May, H., Shalem, D., Rohland, N., Mallick, S., Lazaridis, I., Sarig, R., Stewardson, K., Nordenfelt, S., Patterson, N., et al. (2018). Ancient DNA from Chalcolithic Israel reveals the role of population mixture in cultural transformation. Nat. Commun. *9*, 3336.

S30. de Barros Damgaard, P., Martiniano, R., Kamm, J., Víctor Moreno-Mayar, J., Kroonen, G., Peyrot, M., Barjamovic, G., Rasmussen, S., Zacho, C., Baimukhanov, N., et al. (2018). The first horse herders and the impact of early Bronze Age steppe

S31. Valdiosera, C., Günther, T., Vera-Rodríguez, J.C., Ureña, I., Iriarte, E., Rodríguez-Varela, R., Simões, L.G., Martínez-Sánchez, R.M., Svensson, E.M., Malmström, H., et al. (2018). Four millennia of Iberian biomolecular prehistory illustrate the impact of prehistoric migrations at the far end of Eurasia. Proc. Natl. Acad. Sci. U. S. A. *115*, 3428–3433.

S32. Brace, S., Diekmann, Y., Booth, T.J., van Dorp, L., Faltyskova, Z., Rohland, N., Mallick, S., Olalde, I., Ferry, M., Michel, M., et al. (2019). Ancient genomes indicate population replacement in Early Neolithic Britain. Nat Ecol Evol *3*, 765–771.

S33. González-Fortes, G., Tassi, F., Trucchi, E., Henneberger, K., Paijmans, J.L.A., Díez-Del-Molino, D., Schroeder, H., Susca, R.R., Barroso-Ruíz, C., Bermudez, F.J., et al. (2019). A western route of prehistoric human migration from Africa into the Iberian Peninsula. Proc. Biol. Sci. *286*, 20182288.

S34. Olalde, I., Mallick, S., Patterson, N., Rohland, N., Villalba-Mouco, V., Silva, M., Dulias, K., Edwards, C.J., Gandini, F., Pala, M., et al. (2019). The genomic history of the Iberian Peninsula over the past 8000 years. Science *363*, 1230–1234.

S35. Narasimhan, V.M., Patterson, N., Moorjani, P., Rohland, N., Bernardos, R., Mallick, S., Lazaridis, I., Nakatsuka, N., Olalde, I., Lipson, M., et al. (2019). The formation of human populations in South and Central Asia. Science *365*.

S36. Feldman, M., Fernández-Domínguez, E., Reynolds, L., Baird, D., Pearson, J., Hershkovitz, I., May, H., Goring-Morris, N., Benz, M., Gresky, J., et al. (2019). Late Pleistocene human genome suggests a local origin for the first farmers of central Anatolia. Nat. Commun. *10*, 1218.

S37. Sánchez-Quinto, F., Malmström, H., Fraser, M., Girdland-Flink, L., Svensson, E.M., Simões, L.G., George, R., Hollfelder, N., Burenhult, G., Noble, G., et al. (2019). Megalithic tombs in western and northern Neolithic Europe were linked to a kindred society. Proc. Natl. Acad. Sci. U.

S. A. *116*, 9469–9474.

S38. Scheib, C.L., Hui, R., D'Atanasio, E., Wohns, A.W., Inskip, S.A., Rose, A., Cessford, C., O'Connell, T.C., Robb, J.E., Evans, C., et al. (2019). East Anglian early Neolithic monument burial linked to contemporary Megaliths. Ann. Hum. Biol. *46*, 145–149.

S39. Antonio, M.L., Gao, Z., Moots, H.M., Lucci, M., Candilio, F., Sawyer, S., Oberreiter, V., Calderon, D., Devitofranceschi, K., Aikens, R.C., et al. (2019). Ancient Rome: A genetic crossroads of Europe and the Mediterranean. Science *366*, 708–714.

S40. Cassidy, L.M., Maoldúin, R.Ó., Kador, T., Lynch, A., Jones, C., Woodman, P.C., Murphy, E., Ramsey, G., Dowd, M., Noonan, A., et al. (2020). A dynastic elite in monumental Neolithic society. Nature *582*, 384–388.

S41. Fernandes, D.M., Mittnik, A., Olalde, I., Lazaridis, I., Cheronet, O., Rohland, N., Mallick, S., Bernardos, R., Broomandkhoshbacht, N., Carlsson, J., et al. (2020). The spread of steppe and Iranian-related ancestry in the islands of the western Mediterranean. Nature Ecology & Evolution *4*, 334–345.

S42. Rivollat, M., Jeong, C., Schiffels, S., Küçükkalıpçı, İ., Pemonge, M.-H., Rohrlach, A.B., Alt, K.W., Binder, D., Friederich, S., Ghesquière, E., et al. (2020). Ancient genome-wide DNA from France highlights the complexity of interactions between Mesolithic hunter-gatherers and Neolithic farmers. Sci Adv *6*, eaaz5344.

S43. Ruden, D., Cingolani, P., Patel, V., Coon, M., Nguyen, T., Land, S., and Lu, X. (2012). Using Drosophila melanogaster as a Model for Genotoxic Chemical Mutational Studies with a New Program, SnpSift. Front. Genet. *3*.
